# A bicistronic vector backbone for rapid seamless cloning and chimerization of αβT-cell receptor sequences

**DOI:** 10.1371/journal.pone.0238875

**Published:** 2020-09-09

**Authors:** Korbinian N. Kropp, Tim J. Schäufele, Martina Fatho, Michael Volkmar, Roland Conradi, Matthias Theobald, Thomas Wölfel, Catherine Wölfel

**Affiliations:** 1 Internal Medicine III, University Cancer Center (UCT), Research Center for Immunotherapy (FZI), University Medical Center (UMC) of the Johannes Gutenberg University and German Cancer Consortium (DKTK), Partner Site Frankfurt/Mainz, Germany; 2 Division of Molecular Oncology of Gastrointestinal Tumors, German Cancer Research Center (DKFZ), Heidelberg, Germany; 3 Helmholtz-Institute for Translational Oncology Mainz (HI-TRON Mainz)—a Helmholtz institute by DKFZ, Mainz, Germany; 4 Transfusion Center, University Medical Center (UMC) of the Johannes Gutenberg University, Mainz, Germany; Fudan University, CHINA

## Abstract

To facilitate preclinical testing of T-cell receptors (TCRs) derived from tumor-reactive T-cell clones it is necessary to develop convenient and rapid cloning strategies for the generation of TCR expression constructs. Herein, we describe a pDONR™221 vector backbone allowing to generate Gateway™ compatible entry clones encoding optimized bicistronic αβTCR constructs. It harbors P2A-linked TCR constant regions and head-to-head-oriented recognition sites of the Type IIS restriction enzymes BsmBI and BsaI for seamless cloning of the TCRα and TCRβ V(D)J regions, respectively. Additional well-established TCR optimizations were incorporated to enhance TCR functionality. This included replacing of the human αβTCR constant regions with their codon-optimized murine counterparts for chimerization, addition of a second interchain disulfide bond and arrangement of the TCR chains in the order β-P2A-α. We exemplified the utility of our vector backbone by cloning and functional testing of three melanoma-reactive TCRs in primary human T cells.

## Introduction

Adoptive transfer of *in vitro* expanded tumor infiltrating lymphocytes (TIL) naturally expressing cancer-reactive T-cell receptors (TCR) has yielded promising results in metastatic melanoma [1] and in epithelial cancers [2]. However, this treatment option is confined only to patients, in whom the expansion of functional TIL is successful [3]. This limitation can be overcome by identification and cloning of the αβTCR of tumor-reactive T-cell clones to generate αβTCR-transduced blood-derived T cells for adoptive therapy [4]. αβTCR expression constructs often share optimizations that favor expression of transgenic TCRs and reduce putative mispairing with endogenous α and β TCR chains. These, amongst others, comprise expression of the α and β TCR chains from the same promoter by linking the individual genes with a 2A element [5], arrangement in the order β-2A-α [5, 6], replacement of the human constant domains with their murine counterparts (chimerization) [7] and introduction of a second disulfide bond into the αβTCR constant domain [8, 9].

As of yet, we employed the MultiSite Gateway™ cloning system [10] to assemble bicistronic αβTCR constructs and integrate the optimizations described above. This strategy required about three weeks of hands-on time, mainly due to the need to remove cloning scars between the joined DNA fragments. Here we suggest a TCR-cloning strategy relying on a single universal vector backbone designed to incorporate TCR-specific V(D)J sequences and to generate chimerized and optimized αβTCR chains preferentially pairing with each other. The method described herein follows the Golden Gate cloning approach that capitalizes on the unique feature of Type IIS restriction enzymes [11]. These restriction enzymes cut outside of their recognition site which allows to freely choose the resulting overhangs for seamless and directional cloning. Using this approach, we could assemble chimerized and optimized αβTCR constructs in two to four days and generate TCR expression vectors in four to six days. We validated our approach with three melanoma-reactive αβTCRs retrovirally transduced into primary human T cells. Compared to our previous TCR cloning strategy, we could greatly reduce hands-on time while retaining high cloning efficiency.

## Material and methods

### Cell lines

293FT cells (kindly provided by Thomas Kindler, University Cancer Center Mainz, Germany) and Phoenix amphotropic packaging cells were cultivated in DMEM. COS-7 cells and the previously described human melanoma cell lines SK29-MEL.1 [12] and D05 [13] were cultured in RPMI 1640. Cell-culture media were supplemented with 10% heat-inactivated FCS (Sigma Aldrich, St. Louis, MO, USA) and 1% Penicillin/Streptomycin (Sigma Aldrich) unless stated otherwise.

### T-cell culture

Transgenic T cells were freshly generated by retroviral transduction. T cells were cultured in Panserin (PAN-Biotech, Aidenbach, Germany) supplemented with 10% heat-inactivated human serum (kindly provided by the blood bank of the University Medical Center Mainz), 1% Penicillin/Streptomycin (Sigma Aldrich), rhIL-2 (250 U/mL—600 U/mL; Novartis, Basel, Switzerland). Transgenic T cells were once stimulated with anti-CD3/CD28 beads (Thermo Fisher Scientific, Waltham, MA, USA) on the day after transduction and were then weekly restimulated with irradiated (10,000 Gy) antigen-expressing tumor cells at a stimulator-to-T cell ratio of 1:5. Transgenic T cells were additionally selected with puromycin (1 μg/mL; Sigma Aldrich).

### Cloning and vectors

PCR reactions were set up using the Q5 High-fidelity DNA Polymerase or the *Taq* DNA Polymerase (both NEB, Ipswich, MA, USA). Restriction enzymes (BsmBI, BsaI) and the T4 ligase with appropriate buffers were from NEB and Thermo Fisher Scientific. Where indicated, vectors and PCR products were purified with the Monarch Gel Extraction kit (NEB) or with PCR Clean-up columns (Macherey Nagel, Dueren, Germany). Plasmid preparations were done with the Plasmid kits from Qiagen (Hilden, Germany). 10-beta competent E. coli (NEB) were used for plasmid transformation.

The Gateway™ compatible entry vector pDONR™221 (Thermo Fisher Scientific) was modified by standard molecular cloning techniques as described in the results section. The pMXs-IRES-Puro retroviral expression vector (Cell Biolabs, San Diego, CA, USA) served as destination vector. Following the Gateway™ converter system (Thermo Fisher Scientific) the pMXs-IRES-Puro vector was converted to a Gateway™ compatible destination vector by insertion of the chloramphenicol resistance gene and the ccdB gene flanked by *att*R recognition sites at the multiple cloning site using the XhoI restriction site. In the final destination vector (pMXs-IRES-puro-DEST) the transgene is connected to the puromycin resistance gene by an IRES sequence which allows expression from the same retroviral promotor. Entry vectors were recombined into the final destination vector performing the LR clonase (Thermo Fisher Scientific) reaction.

The retroviral pMP71-PRE plasmid harboring the codon optimized (GeneArt) and chimerized (codon optimized murine constant regions) anti-CDK4^R24C^ TCR derived from the HLA-A*02 restricted T-cell clone 14/35 has previously been described by others [14].

The Firefly luciferase (Fluc) expression plasmid pLenti-EF1a-Pac-T2A-Fluc was generated by replacing the Gaussia luciferase (Gluc) of the pLenti-EF1a-Pac-T2A-Gluc plasmid [15] (kindly provided by Preet Chaudhary, University of Southern California, CA, USA) with the Firefly Luciferase hluc^+^ (Promega, Waltham, MA, USA) via Gibson Assembly (NEB).

Sequencing was performed using the services of Eurofins (Luxembourg, Luxembourg). Sequence analyses were made using Geneious software (Biomatters, Auckland, New Zealand, Version 8.1.9).

### Production of retroviral particles

Retroviral particles were generated as previously described [16]. In brief, Phoenix amphotropic retroviral packaging cells were cotransfected with the helper plasmids pCOLT-GALV and pHIT60 (both 5 μg per culture dish) as well as the respective pMXs expression vector (10 μg per culture dish) using FuGENE6 Transfection Reagent (Promega, Madison, WI, USA) according to the manufacturer´s instructions. The next day, medium was replaced with T-cell culture medium and supernatant was harvested after additional incubation for 16 h by pelleting cellular debris.

### Retroviral transduction of CD4^+^ and CD8^+^ T cells

CD3^+^ T cells were purified from PBMC using the CD3^+^ selection kit (Miltenyi Biotec, Bergisch Gladbach, Germany). PBMC from healthy donors were provided by the blood bank of our institution according to the guidelines of the local ethics committee (Ethics committee of the Medical Association of Rhineland-Palatinate, Mainz, Germany). All donors gave written informed consent. Transduction of PBMC with retroviral vectors was exclusively performed within a genetic engineering facility (registration number: B 11.110) in accordance with the German Genetic Engineering Act. For retroviral transduction freshly prepared CD3^+^ T cells were activated for 2 days with an anti-CD3 antibody (30 ng/mL; clone: OKT3; hybridoma obtained from ATCC, Manassas, VA, USA) and rhIL-2 (600 U/mL). Subsequently, CD3^+^ T cells were spinofected with virus particles (90 min at 2000 rpm) in the presence of Polybrene (4 μg/mL; Sigma Aldrich) and rhIL-2 (600 U/mL), and were further cultivated for 22 h or were left untreated. Thereafter, transgenic T cells were cultured as described above. Where indicated CD4^+^ and CD8^+^ T cells were isolated from transgenic CD3^+^ T cells using the CD4^+^ and CD8^+^ selection kit (Miltenyi Biotec), respectively.

### Flow cytometry

Cells were surface-stained with the following fluorescent-conjugated antibodies: Anti-Vβ1-PE (clone BL37.2, 5 μl), anti-Vβ4-PE (clone WJF24, 5 μl), anti-CD8-FITC (clone B9.11, 2 μl), anti-CD8-PE (clone B9.11, 2 μl) and IgG-PE/IgG-FITC (clone 679.1Mc7, 4 μl) was from Beckman Coulter (Brea, CA, USA) and anti-murine TCR-FITC (clone CL075F, 1 μl) from OriGene (Rockville, MD, USA). Flow cytometry was performed on a FACS Canto II (BD Bioscience, San José, CA, USA) and data was analyzed using FlowJo (BD Bioscience, Version 8.0).

### IFNγ-ELISpot assay

IFNγ-ELISpot-assays were performed as described previously [17]. In brief, 293T or COS-7 cells (20,000 cells/well) were transfected with cDNA encoding HLA-A*02:01. Where indicated, cells were cotransfected with cDNA encoding full length CDK4 harboring the R24C mutation or encoding full-length NY-ESO-1. Transfection was performed directly on ELISpot plates (Millipore, Burlington, MA, USA) using Lipofectamine 2000 (Invitrogen, Carlsbad, CA, USA) according to the manufacturer´s recommendations. Twenty-four hours thereafter transfectants were additionally pulsed with the peptide ACDPHSGHFV (CDK4^R24C^) or SLLMWITQC (NY-ESO-1), where indicated (both synthesized by Dr. Jan-Wouter Drijfhout, University Medical Center Leiden, The Netherlands). Transgenic T cells (5,000–10,000 TCR^+^ cells/well) were then added to (peptide pulsed) transfectants or to freshly seeded melanoma cells (50,000 cells/well). Where indicated, cells were cultured in the presence of the HLA-A*02 blocking antibody MA2.1 at a concentration of 200 μg/mL (clone: MA2.1; hybridoma obtained from ATCC). After 20–24 h incubation ELISpot plates were developed and IFNγ spots were visualized with an ImmunoSpot analyzer (Cellular Technology Limited, Cleveland, OH, USA).

### Cytotoxicity assay

Cytotoxicity of effector T-cells was evaluated performing a bioluminescence-based lysis assay as recently described [18, 19]. To this end SK29-MEL.1 or D05 melanoma cells stably expressing Firefly luciferase (Fluc) were generated by lentiviral transduction. Melanoma cells (10,000 cells/well) were cocultured with effector cells at the indicated effector-to-target ratio in the presence of 0.15 mg/mL D-Luciferin (Biosynth, St. Gallen, Switzerland). Eighteen hours later, relative luminescence units (RLU) were determined using a FluoStar Omega plate reader (BMG Labtech, Ortenberg, Germany) and a 10 s integration time. Spontaneous cell death was measured in wells containing target cells only, maximum cell death was induced by exposure of target cells to Digitonin (Sigma) at a concentration of 30 μg/mL. Lysis was calculated using the following equation: lysis [%] = 100*((spontaneous RLU—test unit RLU) / (spontaneous RLU—maximum RLU)).

## Results

### Structure of the seamless-cloning vector backbone

**Fig 1:**
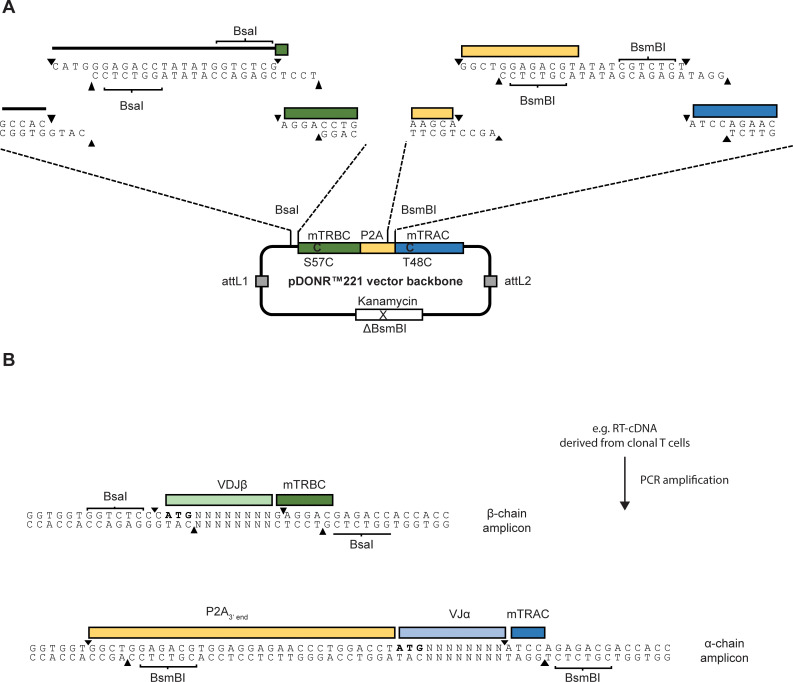
Schematic depiction of the vector backbone for generation of chimerized αβTCR constructs by BsmBI and BsaI restriction. **(A)** The VBB represents a pDONR™221 vector harboring the invariable elements of a chimerized bicistronic αβTCR construct comprising the murine TCR beta and alpha chain constant regions (mTRBC and mTRAC) separated by a P2A element. In addition, two BsaI and BsmBI restriction sites each, are placed in head-to-head orientation at the vector/mTRBC and the P2A/mTRAC junctions, respectively, leading to unique overhangs upon cleavage. A naturally occurring BsmBI-restriction site in the Kanamycin resistance gene has been removed by site-directed mutation. **(B)** The TCRα chain VJ (VJα) and the TCRß chain VDJ (VDJβ) regions of the TCR of choice are flanked during PCR amplification with BsmBI- or BsaI-restriction sites, respectively, to generate appropriate overhangs. PCR fragments are then restricted and ligated into the vector backbone yielding the full-length chimerized αβTCR construct. Spikes indicate cleavage sites of the Type IIS restriction enzymes required for generation of homologous overhangs and removal of non-coding or redundant sequences.

In order to accelerate generation of αβTCR expression constructs, we designed a Gateway™ compatible pDONR™221 entry vector backbone (VBB) for seamless cloning and chimerization of variable αβTCR sequences (summarized in Fig 1). The VBB harbors a bicistronic TCR-expression cassette lacking the TCRα- and TCRβ-variable sequences. The TCR-expression cassette comprises the murine TCRα- and TCRβ-chain constant regions (mTRAC and mTRBC) separated by the ribosomal skipping element P2A and arranged in the order β-P2A-α. mTRAC and mTRBC are codon-optimized and harbor the previously described T48C and S57C mutations, respectively, resulting in an additional interchain disulfide bond [9]. Following the Golden Gate cloning strategy [11], two different Type IIS restriction enzyme recognition sites were placed each in head-to-head orientation at the 5’ end of the mTRBC region (BsaI) and at the P2A-mTRAC junction (BsmBI). Upon plasmid cleavage with BsaI or BsmBI non-compatible overhangs are generated (Fig 1A). These unique overhangs were then used to directionally and scarlessly insert PCR-amplified TCRα- and TCRβ-variable sequences to assemble full length chimerized TCR (cTCR) constructs. To this end the antigen-specific VJα and VDJβ regions are flanked during PCR amplification with BsmBI and BsaI restriction sites, respectively (Fig 1B). Overhangs are designed to allow precise and seamless ligation of the VJα fragment at the P2A/mTRAC junction and of the VDJβ fragment at the 5’ end of the mTRBC region of the VBB. Of note, it is crucial to reconstruct the P2A element via primer design since the 3’ end of P2A is excised upon BsmBI restriction of the VBB due to a naturally occurring BsmBI restriction site within the P2A element. Core primer sequences used to amplify VJα and VDJβ fragments are given in Table 1.

**Table 1:** Primer used to generate TCRα and TCRβ V(D)J fragments.

V(DJ)	Primer	Sequence 5’-3’
TCRα	BsmBI.alpha.for	GGTGGT**GGCT**GGAGACGTGGAGGAGAACCCTGGACCT NNNNNN. . .
		. . .CTCTGC. . .
	BsmBI.alpha.rev	GGTGGTCGTCTCT**GGAT** NNNNNN. . .
TCRβ	BsaI.beta.for	GGTGGTGGTCTCC**CATG** NNNNNN. . .
	BsaI.beta.rev	GGTGGTGGTCTCG**TCCT**C NNNNNN. . .

Type IIS restriction enzyme recognition sites are underlined, unique overhangs generated upon cleavage are shown in boldface type and TCR-specific sequences are indicated by N.

The resulting bicistronic chimerized αβTCR construct can then be subcloned into any Gateway™ compatible expression vector performing the Gateway™ LR clonase reaction for further testing.

### Assembly of bicistronic chimerized αβTCR constructs

We employed our VBB to either assemble αβTCR expression constructs *de novo* using as starting material RT-cDNA transcribed from clonal T cells—the respective TCR sequence had been identified following the methods described by Boria et al. [20] and Birkholz et al. [21]—or we re-constructed αβTCRs that had been previously cloned but e.g. had not been chimerized. Specific primer used to assemble all cTCR expression constructs in this study along with specification of the starting material (RT-cDNA or plasmid DNA) are given in S1 Table.

**Fig 2:**
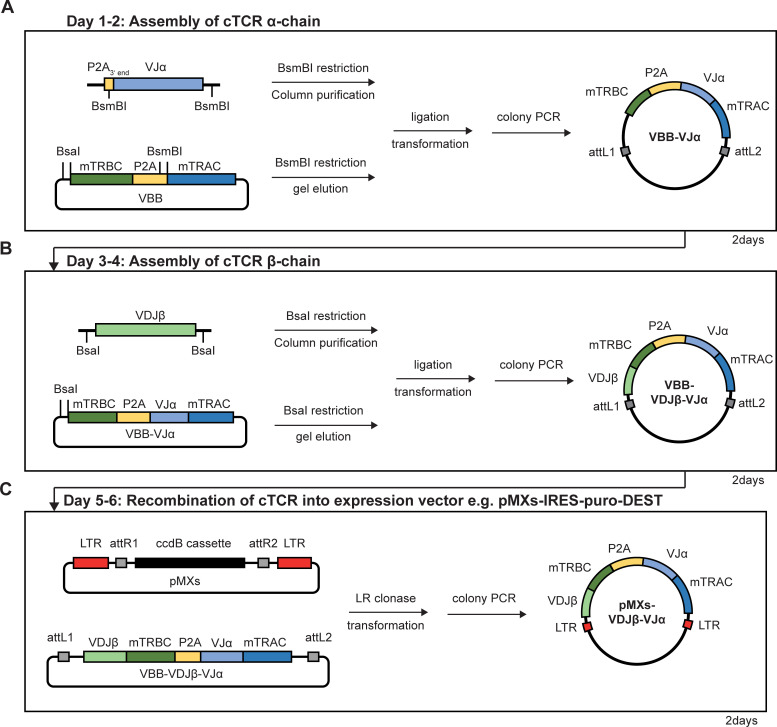
Workflow for generation of cTCR expression constructs using the VBB. **(A)** Day 1–2: The VJα fragment was generated by PCR, column purified, restricted by BsmBI and again column purified. VBB was restricted by BsmBI and gel eluted. Purified VBB and VJα fragments were ligated and the recombined plasmid was transformed. Colony PCR was used to identify clones carrying the insert (VBB-VJα). **(B)** Days 3–4: VDJβ fragment was generated by PCR, column purified, restricted by BsaI and again column purified. Plasmid from VBB-VJα was prepared, restricted by BsaI and gel eluted. Purified VBB-VJα and VDJβ fragments were ligated and the recombined plasmid was transformed. Colony PCR was used to identify clones carrying the insert (VBB-VDJβ-VJα). Days 5–6: Plasmid from VBB-VDJβ-VJα was prepared and recombined into a Gateway compatible expression vector e.g. pMXs-IRES-puro-DEST performing the LR-clonase reaction. Positive clones (pMXs-VDJβ-VJα) were identified by colony PCR.

**Fig 3:**
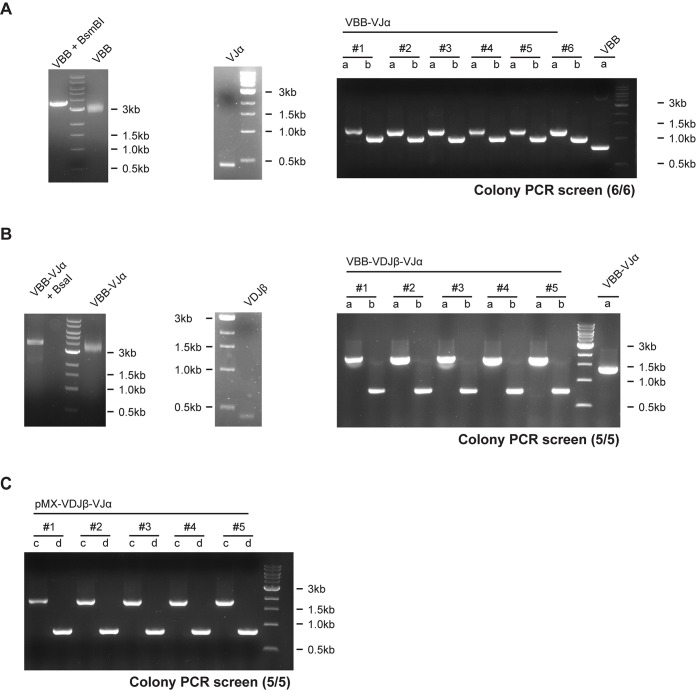
Assembly of a cTCR derived from T-cell clone 55/74. Workflow as shown in Fig 2. **(A)** Left: The VBB was restricted by BsmBI and gel purified. Uncut VBB served as control. Middel: The VJα fragment was amplified by PCR, loaded on a gel to verify correct amplification, column purified and restricted by BsmBI and column purified. Right: Colony PCR was used to screen VBB-VJα positive clones using primer pairs a (M13.for, mTRAC.p670.rev) and b (M13.for, mTRBC.p618.rev). VBB served as control. **(B)** Left: The VBB was restricted by BsaI and gel purified. Uncut VBB-VJα served as control. Middle: The VDJβ fragment was amplified by PCR using the primer given in S1 Table, loaded on a gel to verify correct size, restricted by BsaI and column purified. Right: Colony PCR screen used to identify VBB-VDJβ-VJα positive clones using primer pairs a and b. VBB-VJα served as control. **(C)** LR-clonase reaction was performed to recombine the cTCR construct into our pMXs expression vector. Colony PCR screen used to identify pMXs-VDJβ-VJα clones with primer pairs c (pMXs.for, mTRAC.p670.rev) and d (pMXs.for, mTRBC.p618.rev).

In initial experiments, αβTCR constructs were generated in a two-step process summarized in Fig 2. In the first step the cTCRα chain was assembled (Fig 2A). VJα fragments were flanked with BsmBI recognition sites by PCR, column purified, digested by BsmBI and were again column purified. We then ligated the VJα fragments into BsmBI restricted and gel eluted VBB, transformed the recombined plasmid and verified successful assembly of the cTCRα chain by colony PCR and Sanger sequencing of positive clones. S2 Table summarizes primer used to identify positive transformants via colony PCR and primer used for sequencing. In the second step we assembled the cTCRβ chain following a similar procedure (Fig 2B). After successful assembly, the full-length cTCR construct was then subcloned into the Gateway™ compatible expression vector pMX-IRES-puro-DEST that had been generated as described in the Material and Methods section (Fig 2C). Using this approach cloning of the final cTCR expression vector was usually achieved within only six days. An exemplary result is shown in Fig 3 showing assembly of a cTCR expression vector using as template plasmid DNA harboring a non-optimized αβTCR derived from the tumor-reactive T-cell clone 55/74. Assembly of both the cTCRα and cTCRβ chain was highly efficient with 6/6 and 5/5 positive clones, respectively, as determined by colony PCR (Fig 3A and 3B). As expected subcloning of the full-length cTCR construct into the Gateway™ compatible pMXs-IRES-puro-DEST expression vector was also highly efficient (Fig 3C). Using this two-step approach, we successfully constructed eight different cTCRs using as starting material either RT-cDNA (n = 2) or plasmid DNA (n = 6). The average efficiency (n = 8) for assembly of the cTCRα chain and cTCRβ chain as determined by colony PCR was 98% and 84%, respectively (S3 Table).

In an effort to further reduce hands-on time and material usage we combined BsaI and BsmBI restriction of both the VBB and the column-purified V(D)J-encoding PCR products as well as ligation into the vector backbone in a one-step, one-vessel restriction/ligation reaction. An example of successful *de novo* assembly of cTCR expression constructs from RT-cDNA derived from the melanoma reactive T-cell clones 2C/406 and 5C/169 using the one-step approach is shown in S1 Fig. From the respective 8 analyzed colonies 3 (5C/169) and 8 (2C/406) colonies were screened positive for the full-length cTCR construct corresponding to a cloning efficiency of 38% and 100% respectively. The average cloning efficiency of the one-step approach (n = 6) was 46% as summarized in S3 Table. Of note, the lowest cloning efficiency was observed in the presence of additional Type IIS restriction enzyme recognition sites within the V(D)J-encoding PCR products resulting in only 9% (T-cell clone 4/134) and 15% (T-cell clone 4/76) positive colonies when performing the one-step approach. Collectively these results suggest that although the one-step approach allows to generate a full-length cTCR construct within only 2 days (and the final expression vector within 4 days) the cloning efficiency is drastically reduced as compared to the standard two-step approach as shown in Fig 2.

### Generation of transgenic T cells expressing cTCRs using the seamless-cloning vector backbone

To functionally validate our αβTCR cloning vector backbone we chose the well-characterized CD8^+^ T-cell clone 14/35 directed against mutant CDK4 (R24C) that has originally been established via an autologous mixed lymphocyte-tumor culture (MLTC) using the CDK4^R24C^-positive melanoma-cell line SK29-MEL.1 as stimulator of blood-derived T cells [12]. Its HLA-A*02-restricted αβTCR has been cloned by others into the retroviral expression vector pMP71-PRE [14]. In addition, we cloned two αβTCR *de novo* using as starting material RT-cDNA transcribed from RNA derived from two HLA-A*02 restricted NY-ESO-1 specific CD8^+^ T cell clones. The respective T cell clones 4/76 and 4/134 had been established in our group via MLTCs using the HLA-A*02^+^ NY-ESO-1^+^ melanoma cell line D05 as stimulator of blood-derived T cells and recognized the same NY-ESO-1 peptide (SLLMWITQC). Using the primer given in S1 Table and the pMP71-PRE plasmid harboring the anti-CDK4^R24C^/A2 αβTCR construct or RT-cDNA derived from the T cell clones 4/76 or 4/134 as template we PCR-amplified the respective TCRα and TCRβ V(D)J regions and generated pDONR™221 entry clones as described above. Subsequently, the final bicistronic αβTCR constructs were recombined into the retroviral expression vector pMXs-IRES-Puro-DEST by performing the Gateway™ LR-clonase reaction.

**Fig 4:**
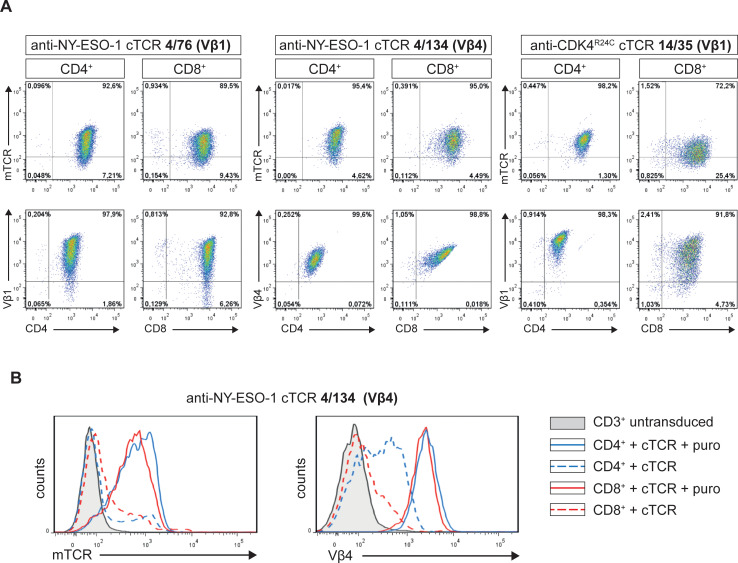
Expression of cTCRs in CD4^+^ and CD8^+^ T cells. (A) CD4^+^ and CD8^+^ T cells were retrovirally transduced with the indicated cTCR and expanded in the presence of puromycin. Flow cytometry analysis depicting expression of the indicated cTCR in CD4^+^ and CD8^+^ T cells. (B) CD4^+^ and CD8^+^ T cells were retrovirally transduced with the anti-NY-ESO-1/A2 cTCRs 4/134 and were expanded in the presence or absence of puromycin. Flow cytometry analysis showing expression of cTCR in CD4^+^ and CD8^+^ T cells as compared to untransduced CD3^+^ T cells. (A-B) Antibodies directed against the murine TCR constant region (mTCR) or against the variable region (Vβ) of the respective cTCR were used.

After assembly of the respective retroviral expression constructs, we transduced the anti-NY-ESO-1/A2 cTCRs 4/76 and 4/134 and the anti-CDK4^R24C^/A2 cTCR 14/35 into CD3^+^ T cells. After short term expansion in the presence of puromycin, CD4^+^ and CD8^+^ populations were enriched to high purity (above 95%) by MACS isolation. As shown in Fig 4A we observed high expression levels of all three cTCRs in both CD4^+^ and CD8^+^ T cells. Expression of cTCRs was verified with both a mAB specific for the Vβ chain of the respective cTCR and a mAB recognizing the murine TCR constant region, which gave similar results. As shown in Fig 4B puromycin selection was crucial in order to obtain high expression levels of cTCRs. Of note, additional cTCRs (n = 9) cloned in this study had been successfully expressed in primary T cells with an average expression level of 80% on day 4–17 after transduction (S4 Table).

**Fig 5:**
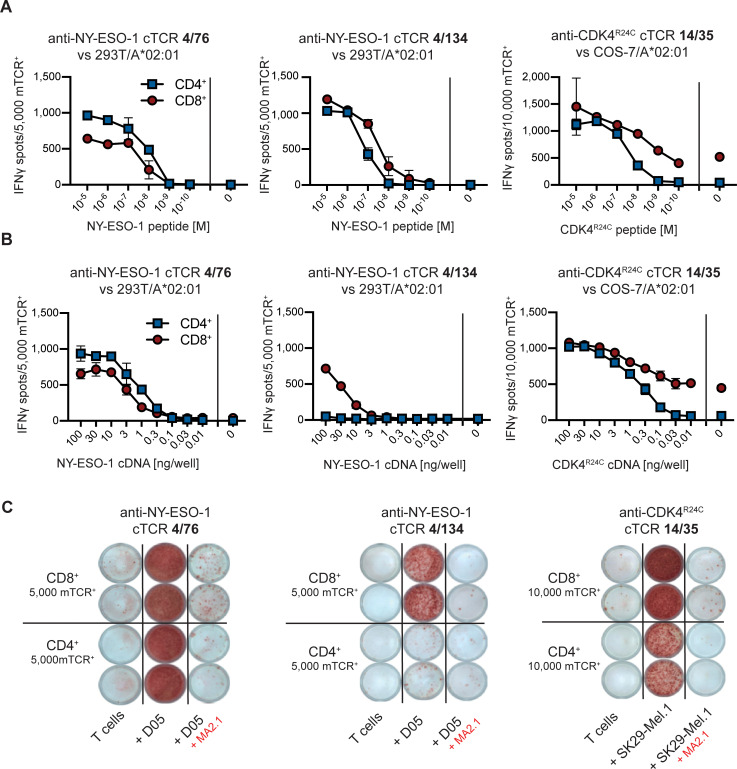
cTCR^+^ CD4^+^ and CD8^+^ T cells specifically recognize antigen expressing target cells. IFNγ production of CD4^+^ and CD8^+^ T cell transduced with the indicated cTCR as determined in 24 h ELISpot assays. cTCR expression was analyzed prior to testing and results were used to correct for the amount of cTCR^+^ cells within different T cell populations applied to functional tests. **(A-C)** IFNγ production after incubation of T cells with HEK-293T or COS-7 cells transiently transfected with HLA-A*02:01 and **(A)** loaded with titrated amounts of the indicated peptide or **(B)** cotransfected with titrated amounts of cDNA encoding the indicated antigen. **(C)** IFNγ production after incubation of T cells with either no target cells or with NY-ESO-1^+^ HLA-A*02^+^ D05 cells or CDK4^R24C+^ HLA-A*02^+^ SK29-MEL.1 cells in the presence or absence of the HLA-A*02 blocking antibody MA2.1. Data points represent mean values of doublets ± standard deviation.

Next, we analyzed specificity and functionality of CD4^+^ and CD8^+^ T cells transduced with the anti-NY-ESO-1/A2 cTCRs 4/76 and 4/134 or the anti-CDK4^R24C^/A2 cTCR 14/35 in 24 h IFNγ-ELISpot assays. In a first step, redirected CD4^+^ and CD8^+^ T cells were incubated with 293T or COS-7 cells transfected with HLA-A*02 and loaded with titrated amounts of the NY-ESO-1 peptide (SLLMWITQC) or the CDK4^R24C^ peptide (ACDPHSGHFV), respectively (Fig 5A). Analysis of the anti-NY-ESO-1 specific cTCRs and the anti-CDK4^R24C^ cTCR revealed a similar dose dependent functionality in both CD4^+^ and CD8^+^ T cells. To further analyze recognition of endogenously processed antigen, 293T or COS-7 cells were transfected with HLA-A*02 and were cotransfected with titrated amounts of NY-ESO-1 or CDK4^R24C^ cDNA, respectively (Fig 5B). Interestingly, CD8^+^ 4/134 cTCR-transduced T cells recognized NY-ESO-1 cDNA transfected targets while no response was observed with CD4^+^ T cells expressing the same cTCR. In contrast, both CD4^+^ and CD8^+^ T cells transduced with the 14/35-cTCR or with the 4/76-cTCR displayed similar responses against antigen-cDNA transfected target cells. These results were also mirrored when using as targets HLA-A*02^+^ melanoma cell lines that naturally expressed NY-ESO-1 (D05) or harbored the CDK4^R24C^ mutation (SK29-Mel.1). The response of 4/76 cTCR-transduced T cells against D05 cells and of 14/35 cTCR-transduced T cells against SK29-MEL.1 was similar for CD4^+^ and CD8^+^ T cells (Fig 5C). In contrast CD4^+^ 4/134 cTCR-transduced T cells did not recognize D05 cells despite clear recognition by the respective CD8^+^ population. Addition of an HLA-A*02 blocking antibody (MA2.1) confirmed MHC I-restriction of target recognition.

**Fig 6:**
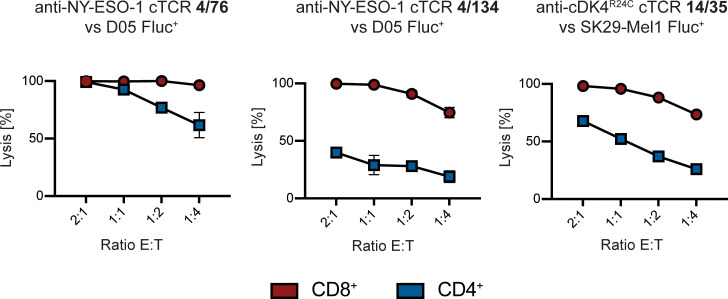
Lytic activity of cTCR^+^ CD4^+^ and CD8^+^ T cells. Cytotoxicity of T cells transduced with the indicated cTCR as determined by a 18 h-bioluminescence assay. cTCR expression was analyzed prior to testing and results were used to correct for the amount of cTCR^+^ cells within different T cell populations applied to functional tests. Target cells were either Firefly luciferase expressing NY-ESO-1^+^ HLA-A*02^+^ D05 cells or CDK4^R24C+^ HLA-A*02^+^ SK29-Mel.1 cells as indicated. Data points represent mean values of triplicates ± standard deviation.

Finally, we analyzed whether cTCR-transgenic T cells also elicit target cell lysis in a bioluminescence-based cytotoxicity assay (Fig 6). D05 and SK29-MEL.1 cells stably expressing Firefly Luciferase were cocultured with redirected CD4^+^ or CD8^+^ transgenic T cells. Analysis of the bioluminescence signal after 18 h essentially mirrored the results shown before. Redirected CD8^+^ T cells showed strong cytotoxicity against antigen positive target cells. CD4^+^ T cells transduced with the same cTCRs were also able to eliminate target cells but to a much lower extent. Of note, and as expected from the results shown before, cytotoxicity of CD4^+^ T cell transduced with the 4/134 cTCR against NY-ESO-1^+^ HLA-A*02^+^ D05 melanoma cells was considerably lower as compared to CD4^+^ T cell transduced with the 4/76 cTCR.

Collectively, we cloned seven αβTCR *de novo* using as starting material RT-cDNA transcribed from as little as 30 ng of RNA derived from clonal T cells. The respective TCR variable regions had been identified following the methods described by Boria et al. [20] and Birkholz et al. [21] as already stated above. Furthermore, we re-constructed six αβTCRs that had been previously cloned but had not been chimerized or had been cloned in the order α-P2A-β. Compared to the parental T-cell clones or the non-optimized αβTCR we observed no loss of function and specificity (not shown).

## Discussion

We report on a vector backbone for the generation of αβTCR constructs. It harbors the uniform components of a bicistronic chimerized αβTCR construct. TCR-specific V(D)J-regions are ligated into this backbone using Type IIS restriction enzymes to assemble full length αβTCR chains. This approach is based on the Golden Gate cloning method [11]. A similar strategy has been applied by others for the same purpose [22, 23], but the vector backbone presented herein displays several unique and distinct features of additional value.

Hu et al. generated full-length TCR constructs by Golden Gate cloning, but ligated only the hypervariable CDR3 region into components of an extensive vector backbone library, members of which each encode one of the different TCR α and β variable sequences [22]. In contrast, our TCR cloning approach is based on a single, uniform vector backbone, which harbors only the TCR constant domains. Although this requires to ligate the complete, and compared to the sole CDR3 region, considerably longer TCR V(D)J sequences into the vector backbone, there is no need to generate and maintain a plasmid library covering all TCR α and β variable sequences. More similar to our approach, Coren et al. generated vector backbones encoding either human or macaque TCR constant regions with head-to-head oriented recognition sites for the Type IIS restriction enzyme BbsI at the vector/TRAC and the P2A/TRBC junctions [23]. TCR α and β variable sequences were flanked with BbsI recognition sites and appropriate overhangs during PCR amplification, and were seamlessly ligated into the vector backbone for assembly of human/macaque αβTCR constructs [23]. While the vector backbones of both groups represent final expression vectors, we first assembled pDONR™221 entry vectors and, in a second step, recombined the final αβTCR construct into a Gateway™ compatible expression vector. Although this adds an additional cloning step, our approach integrates the convenient Gateway™ cloning system therefore allowing to use different (Gateway™ compatible) expression vectors. Of note, several different expression vectors used to study TCR-based therapy have already been converted to Gateway™ compatibility [24, 25].

Another distinctive feature of our backbone plasmid is the usage of two different Type IIS restriction enzymes, BsmBI and BsaI, for the assembly of the TCRα and β chain, respectively. This offers advantages when variable TCR regions of interest harbor a BsmBI or BsaI restriction site. E.g, if a naturally occurring BsaI restriction site would be present in the TCRα VJ-sequence of interest, the two-step cloning procedure would be chosen to start with BsaI-mediated assembly of the TCRβ chain (and vice versa). This is particularly important considering gel-purification of the premature construct after assembly of the first TCR chain and subsequent restriction to circumvent purification of two fragments. When the same Type IIS restriction site (BsmBI or BsaI) is present in both subcloned TCRα and β fragments we would suggest to remove at least one restriction sites e.g. by site-directed mutation or in fact purify two vector backbone fragments.

When performing our standard cTCR assembly approach (summarized in Fig 2) we did observe a very high cloning efficiency that was considerably lower when combining both restriction/ligation of both TCR α and β chains as shown in S1 Fig. This difference might be a result of either the purification steps implemented in our standard protocol or could be due to more efficient ligation of only two fragments when assembling the TCR α and β chains separately. Interestingly, in the presence of internal Type IIS restriction sites in the subcloned fragments, we observed a drastically reduced cloning efficiency when performing restriction/ligation in the same vessel. This is presumably due to continuous restriction and relegation of the assembled vector as also observed by others [11, 23]. A strategy to circumvent this problem is performing restriction and ligation steps sequentially in the same vessel, e.g., by heat inactivation of the restriction enzyme [11].

Our vector backbone integrates many of the optimizations known to enhance transgenic TCR expression and functionality, i.e. (i) codon optimization, (ii) β-P2A-α order, (iii) an additional interchain disulfide bond and (iv) chimerization. Codon optimization is known to positively regulate transcription [26], while the β-P2A-α order is considered to avoid premature degradation of the TCR α chain [5]. Chimerization of TCRs [7] and addition of an interchain disulfide bond [8, 9] have been shown to greatly enhance T-cell reactivity, which was mainly attributed to enhanced TCR expression as a result of preferential pairing of modified TCRs in the presence of endogenous human TCRs. Moreover, it has been shown that chimerization (alike minimal murinization) of TCRs not only increases transgenic TCR expression with regard to percentage and mean fluorescence intensity (MFI) but also leads to enhanced TCR/CD3 stability [7, 27]. It was suggested that this, independently from TCR expression, enhances transgenic TCR functionality although the underlying mechanisms are not yet fully understood [27]. The TCR modifications provided by our backbone vector are especially useful to prevent mispairing with endogenous TCR chains. Autoreactivity of mispaired TCR is a major safety concern in current clinical trials on TCR transfer [28]. Chimerization of transgenic TCRs is one of the safety measures applied, although induction of xenogenic immune reactions is a concern when using murine sequences. However, in a pivotal human trial conducted with fully murine TCRs, cancer regression was observed and no adverse events were attributed to the murine sequences [29]. Although immune responses against murine TCR components have been detected, no impact on clinical outcome has been described [30]. Nevertheless, it has been suggested that a minimal murinization may reduce the risk of immunogenicity [27, 31].

Our functional data confirmed that the vector backbone can be reliably used to express cTCRs in primary T cells and that redirected T cells are functional in a clear HLA-restricted and antigen-specific manner. Functionality of the MHC I-dependent cTCRs analyzed in this study was not restricted to transgenic CD8^+^ T cells but was also observed upon expression in CD4^+^ T cells. This is in line with several other reports showing that CD4^+^ T cells transduced with MHC I-restricted TCRs recognize the respective peptide/MHC I complex and display cytotoxic activity both in vitro [32] as well as in vivo [33]. The ability of CD4^+^ T cells transduced with an MHC I-restricted TCR to circumvent the CD8-coreceptor may resemble a measure of TCR avidity [32]. Along this line another study described CD8-independent function only upon transfer of a highly peptide/MHC I-avid TCR while low avidity MHC-I restricted TCRs did not function in CD4^+^ T cells [34]. Our functional data on the A*02/NY-ESO-1-specific cTCRs derived from T cell clone 4/76 and 4/134 thus suggest a higher avidity of the TCR derived from clone 4/76. In addition, these results implicate the CD8 molecule as an enhancer of functional avidity of MHC I-restricted TCRs as also suggested by others [35].

In conclusion, the TCR-cloning strategy described herein allows to rapidly generate optimized αβTCR expression constructs. The TCR optimizations integrated in the vector backbone are known to enhance transgenic TCR expression and functionality in part by preventing formation of mispaired TCRs. The vector backbone presented herein might therefore represent a useful tool for the preclinical investigation of human αβTCRs in particular when using primary T cells expressing endogenous TCRs as effector cells. Collectively our results demonstrate the utility and versatility of our vector backbone for rapid cloning and simultaneous optimization of functional αβTCR.

## Supporting information

S1 Table Primer used to amplify VJα and VDJβ fragments of all cTCR constructs generated in this study. Antigen specificity and HLA-restriction of the respective TCR is given where known. ^a^T cell clones 5C/169 and 2C/406 express an identical TCR and are derived from the same MLTC. ^b^The sequence 5’ of the BsaI restriction site of primer BsaI_BC44.22_VDJβ.rev has been modified to prevent hairpin formation. Template during PCR was either plasmid DNA or RT-cDNA derived from clonal T cells. nd = not determined.
(XLSX)

S2 Table Primer sequences for colony PCR and Sanger sequencing. (XLSX)

S3 Table Cloning efficiencies using the standard two-step and the one-step cloning approach. Number of positive clones were determined by colony PCR. SD = Standard deviation.
(XLSX)

S4 Table Transduction efficiencies. Summary of all transgenic T cells that were generated by retroviral transduction of TCRs assembled using the seamless-cloning approach. Shown are the percentage of mTCR^+^ clones among the indicated transduced T-cell population as determined by FACS analysis. SD = Standard deviation.
(XLSX)

S1 Fig Assembly of cTCR expression constructs using the one-step cloning approach. (A) Workflow of the one-step cTCR assembly approach. Unrestricted, column purified VJα and VDJβ PCR fragments, the unrestricted vector backbone, BsaI and BsmBI, T4 ligase and T4 ligase buffer were mixed and 30 cycles of restriction/ligation were performed (5 min at 37°C, 5 min at 16°C). (B) VJα and VDJβ fragments were amplified from RT-cDNA derived from the indicated T cell clones using the primer given in S1 Table, loaded on a gel to verify correct amplification and were column purified. Then one-step assembly was performed and recombined plasmids were transformed. (C) Colony PCR was used to identify VBB-VDJβ-VJα positive clones using primer pairs a (M13.for, mTRAC.p670.rev) and b (M13.for, mTRBC.p618.rev). (C) LR-clonase reaction was performed to recombine the cTCR construct into our pMXs-IRES-puro-DEST expression vector. Colony PCR screen was used to identify pMXs-VDJβ-VJα clones using primer pairs c (pMXs.for, mTRAC.p670.rev) and d (pMXs.for, mTRBC.p618.rev).
(EPS)

S1 Raw images (PDF)

## References

[1] Rosenberg SA, Yang JC, Sherry RM, Kammula US, Hughes MS, Phan GQ, et al Durable complete responses in heavily pretreated patients with metastatic melanoma using T-cell transfer immunotherapy. Clinical cancer research: an official journal of the American Association for Cancer Research. 2011;17(13):4550–7. Epub 2011/04/19. 10.1158/1078-0432.CCR-11-0116 21498393 PMC3131487

[2] Tran E, Turcotte S, Gros A, Robbins PF, Lu YC, Dudley ME, et al Cancer immunotherapy based on mutation-specific CD4+ T cells in a patient with epithelial cancer. Science. 2014;344(6184):641–5. Epub 2014/05/09. 10.1126/science.1251102 . 24812403 PMC6686185

[3] Goff SL, Smith FO, Klapper JA, Sherry R, Wunderlich JR, Steinberg SM, et al Tumor infiltrating lymphocyte therapy for metastatic melanoma: analysis of tumors resected for TIL. J Immunother. 2010;33(8):840–7. Epub 2010/09/16. 10.1097/CJI.0b013e3181f05b91 20842052 PMC6322671

[4] Moore T, Wagner CR, Scurti GM, Hutchens KA, Godellas C, Clark AL, et al Clinical and immunologic evaluation of three metastatic melanoma patients treated with autologous melanoma-reactive TCR-transduced T cells. Cancer Immunol Immunother. 2018;67(2):311–25. Epub 2017/10/21. 10.1007/s00262-017-2073-0 . 29052782 PMC5935006

[5] Leisegang M, Engels B, Meyerhuber P, Kieback E, Sommermeyer D, Xue SA, et al Enhanced functionality of T cell receptor-redirected T cells is defined by the transgene cassette. J Mol Med (Berl). 2008;86(5):573–83. Epub 2008/03/13. 10.1007/s00109-008-0317-3 . 18335188

[6] Okamoto S, Amaishi Y, Goto Y, Ikeda H, Fujiwara H, Kuzushima K, et al A Promising Vector for TCR Gene Therapy: Differential Effect of siRNA, 2A Peptide, and Disulfide Bond on the Introduced TCR Expression. Mol Ther Nucleic Acids. 2012;1:e63 Epub 2012/12/20. 10.1038/mtna.2012.52 23250361 PMC3528300

[7] Cohen CJ, Zhao Y, Zheng Z, Rosenberg SA, Morgan RA. Enhanced antitumor activity of murine-human hybrid T-cell receptor (TCR) in human lymphocytes is associated with improved pairing and TCR/CD3 stability. Cancer research. 2006;66(17):8878–86. 10.1158/0008-5472.CAN-06-1450 16951205 PMC2147082

[8] Cohen CJ, Li YF, El-Gamil M, Robbins PF, Rosenberg SA, Morgan RA. Enhanced antitumor activity of T cells engineered to express T-cell receptors with a second disulfide bond. Cancer research. 2007;67(8):3898–903. 10.1158/0008-5472.CAN-06-3986 17440104 PMC2147081

[9] Kuball J, Dossett ML, Wolfl M, Ho WY, Voss RH, Fowler C, et al Facilitating matched pairing and expression of TCR chains introduced into human T cells. Blood. 2007;109(6):2331–8. Epub 2006/11/04. 10.1182/blood-2006-05-023069 17082316 PMC1852191

[10] Cheo DL, Titus SA, Byrd DR, Hartley JL, Temple GF, Brasch MA. Concerted assembly and cloning of multiple DNA segments using in vitro site-specific recombination: functional analysis of multi-segment expression clones. Genome Res. 2004;14(10B):2111–20. Epub 2004/10/19. 10.1101/gr.2512204 15489333 PMC528927

[11] Engler C, Kandzia R, Marillonnet S. A one pot, one step, precision cloning method with high throughput capability. PloS one. 2008;3(11):e3647 Epub 2008/11/06. 10.1371/journal.pone.0003647 18985154 PMC2574415

[12] Wolfel T, Hauer M, Schneider J, Serrano M, Wolfel C, Klehmann-Hieb E, et al A p16INK4a-insensitive CDK4 mutant targeted by cytolytic T lymphocytes in a human melanoma. Science. 1995;269(5228):1281–4. Epub 1995/09/01. . 7652577 10.1126/science.7652577

[13] Pavey S, Johansson P, Packer L, Taylor J, Stark M, Pollock PM, et al Microarray expression profiling in melanoma reveals a BRAF mutation signature. Oncogene. 2004;23(23):4060–7. Epub 2004/03/30. 10.1038/sj.onc.1207563 . 15048078

[14] Leisegang M, Kammertoens T, Uckert W, Blankenstein T. Targeting human melanoma neoantigens by T cell receptor gene therapy. J Clin Invest. 2016;126(3):854–8. Epub 2016/01/26. 10.1172/JCI83465 26808500 PMC4767365

[15] Matta H, Gopalakrishnan R, Choi S, Prakash R, Natarajan V, Prins R, et al Development and characterization of a novel luciferase based cytotoxicity assay. Sci Rep. 2018;8(1):199 Epub 2018/01/11. 10.1038/s41598-017-18606-1 29317736 PMC5760659

[16] Kessels HW, Wolkers MC, van den Boom MD, van der Valk MA, Schumacher TN. Immunotherapy through TCR gene transfer. Nature immunology. 2001;2(10):957–61. Epub 2001/09/29. 10.1038/ni1001-957 . 11577349

[17] Lennerz V, Fatho M, Gentilini C, Frye RA, Lifke A, Ferel D, et al The response of autologous T cells to a human melanoma is dominated by mutated neoantigens. Proceedings of the National Academy of Sciences of the United States of America. 2005;102(44):16013–8. Epub 2005/10/26. 10.1073/pnas.0500090102 16247014 PMC1266037

[18] Mensali N, Myhre MR, Dillard P, Pollmann S, Gaudernack G, Kvalheim G, et al Preclinical assessment of transiently TCR redirected T cells for solid tumour immunotherapy. Cancer Immunol Immunother. 2019;68(8):1235–43. Epub 2019/06/20. 10.1007/s00262-019-02356-2 31214732 PMC6682583

[19] Mestermann K, Giavridis T, Weber J, Rydzek J, Frenz S, Nerreter T, et al The tyrosine kinase inhibitor dasatinib acts as a pharmacologic on/off switch for CAR T cells. Sci Transl Med. 2019;11(499). Epub 2019/07/05. 10.1126/scitranslmed.aau5907 . 31270272 PMC7523030

[20] Boria I, Cotella D, Dianzani I, Santoro C, Sblattero D. Primer sets for cloning the human repertoire of T cell Receptor Variable regions. BMC Immunol. 2008;9:50 Epub 2008/09/02. 10.1186/1471-2172-9-50 18759974 PMC2551579

[21] Birkholz K, Hofmann C, Hoyer S, Schulz B, Harrer T, Kampgen E, et al A fast and robust method to clone and functionally validate T-cell receptors. J Immunol Methods. 2009;346(1–2):45–54. Epub 2009/05/12. 10.1016/j.jim.2009.05.001 . 19427315

[22] Hu Z, Anandappa AJ, Sun J, Kim J, Leet DE, Bozym DJ, et al A cloning and expression system to probe T-cell receptor specificity and assess functional avidity to neoantigens. Blood. 2018;132(18):1911–21. Epub 2018/08/29. 10.1182/blood-2018-04-843763 30150207 PMC6213317

[23] Coren LV, Jain S, Trivett MT, Ohlen C, Ott DE. Production of retroviral constructs for effective transfer and expression of T-cell receptor genes using Golden Gate cloning. Biotechniques. 2015;58(3):135–9. Epub 2015/03/12. 10.2144/000114265 25757546 PMC4827251

[24] Walchli S, Loset GA, Kumari S, Johansen JN, Yang W, Sandlie I, et al A practical approach to T-cell receptor cloning and expression. PloS one. 2011;6(11):e27930 Epub 2011/12/02. 10.1371/journal.pone.0027930 22132171 PMC3221687

[25] Koste L, Beissert T, Hoff H, Pretsch L, Tureci O, Sahin U. T-cell receptor transfer into human T cells with ecotropic retroviral vectors. Gene Ther. 2014;21(5):533–8. Epub 2014/04/04. 10.1038/gt.2014.25 . 24694535

[26] Scholten KB, Kramer D, Kueter EW, Graf M, Schoedl T, Meijer CJ, et al Codon modification of T cell receptors allows enhanced functional expression in transgenic human T cells. Clin Immunol. 2006;119(2):135–45. Epub 2006/02/07. 10.1016/j.clim.2005.12.009 . 16458072

[27] Sommermeyer D, Uckert W. Minimal amino acid exchange in human TCR constant regions fosters improved function of TCR gene-modified T cells. J Immunol. 2010;184(11):6223–31. Epub 2010/05/21. 10.4049/jimmunol.0902055 . 20483785

[28] Govers C, Sebestyen Z, Coccoris M, Willemsen RA, Debets R. T cell receptor gene therapy: strategies for optimizing transgenic TCR pairing. Trends Mol Med. 2010;16(2):77–87. Epub 2010/02/04. 10.1016/j.molmed.2009.12.004 . 20122868

[29] Johnson LA, Morgan RA, Dudley ME, Cassard L, Yang JC, Hughes MS, et al Gene therapy with human and mouse T-cell receptors mediates cancer regression and targets normal tissues expressing cognate antigen. Blood. 2009;114(3):535–46. Epub 2009/05/20. 10.1182/blood-2009-03-211714 19451549 PMC2929689

[30] Davis JL, Theoret MR, Zheng Z, Lamers CH, Rosenberg SA, Morgan RA. Development of human anti-murine T-cell receptor antibodies in both responding and nonresponding patients enrolled in TCR gene therapy trials. Clinical cancer research: an official journal of the American Association for Cancer Research. 2010;16(23):5852–61. Epub 2010/12/09. 10.1158/1078-0432.CCR-10-1280 21138872 PMC3058233

[31] Bialer G, Horovitz-Fried M, Ya'acobi S, Morgan RA, Cohen CJ. Selected murine residues endow human TCR with enhanced tumor recognition. J Immunol. 2010;184(11):6232–41. Epub 2010/04/30. 10.4049/jimmunol.0902047 . 20427762

[32] Morgan RA, Dudley ME, Yu YY, Zheng Z, Robbins PF, Theoret MR, et al High efficiency TCR gene transfer into primary human lymphocytes affords avid recognition of melanoma tumor antigen glycoprotein 100 and does not alter the recognition of autologous melanoma antigens. J Immunol. 2003;171(6):3287–95. Epub 2003/09/10. 10.4049/jimmunol.171.6.3287 12960359 PMC2248799

[33] Morris EC, Tsallios A, Bendle GM, Xue SA, Stauss HJ. A critical role of T cell antigen receptor-transduced MHC class I-restricted helper T cells in tumor protection. Proceedings of the National Academy of Sciences of the United States of America. 2005;102(22):7934–9. Epub 2005/05/24. 10.1073/pnas.0500357102 15908507 PMC1142362

[34] Kuball J, Schmitz FW, Voss RH, Ferreira EA, Engel R, Guillaume P, et al Cooperation of human tumor-reactive CD4+ and CD8+ T cells after redirection of their specificity by a high-affinity p53A2.1-specific TCR. Immunity. 2005;22(1):117–29. Epub 2005/01/25. 10.1016/j.immuni.2004.12.005 . 15664164

[35] Xue SA, Gao L, Ahmadi M, Ghorashian S, Barros RD, Pospori C, et al Human MHC Class I-restricted high avidity CD4(+) T cells generated by co-transfer of TCR and CD8 mediate efficient tumor rejection in vivo. Oncoimmunology. 2013;2(1):e22590 Epub 2013/03/14. 10.4161/onci.22590 23483821 PMC3583927

