# Supplementary material for: A bicistronic vector backbone for rapid seamless cloning and chimerization of αβT-cell receptor sequences
Source: PLoS One. 2020 Sep 9;15(9):e0238875. doi: 10.1371/journal.pone.0238875 (PMC7480877; doi:10.1371/journal.pone.0238875)
Supplement: S1 Raw images — (PDF) [file pone.0238875.s006.pdf]

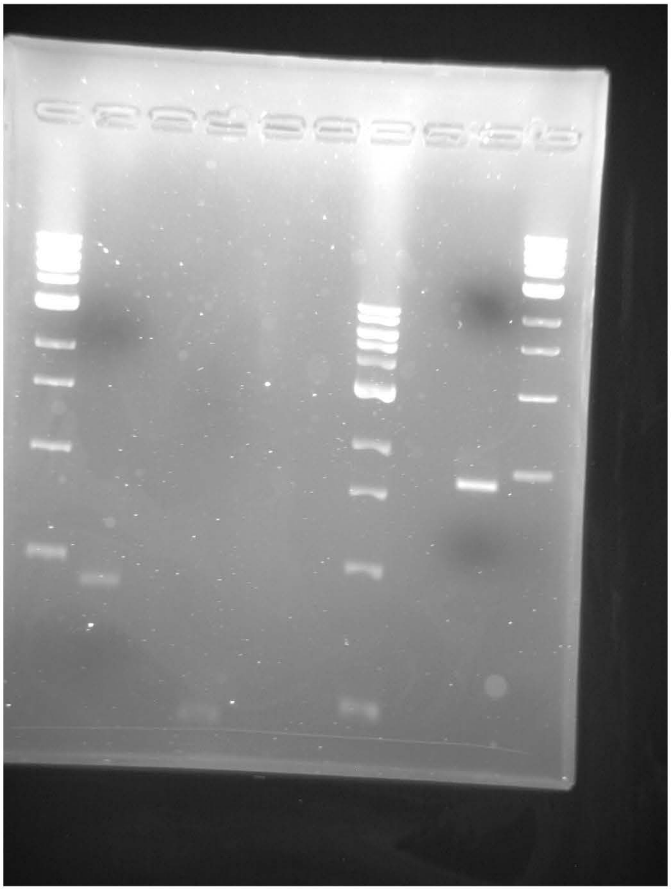

Raw gel image Fig 3A (middle panel) and Fig 3B (middle panel)

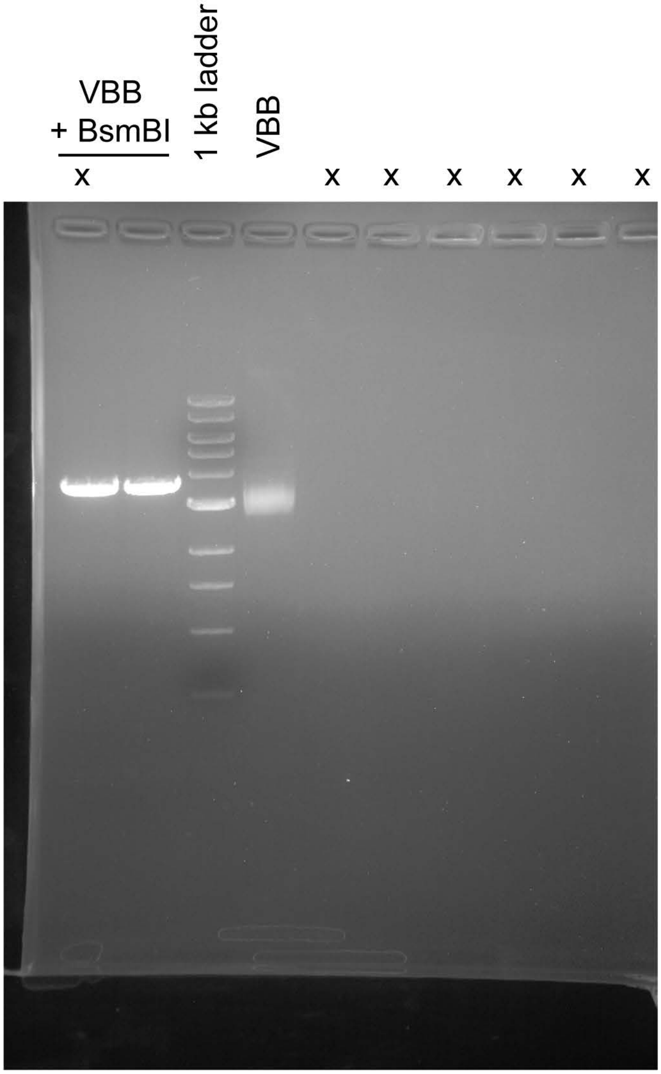

Raw gel image Fig 3A (left pannel)

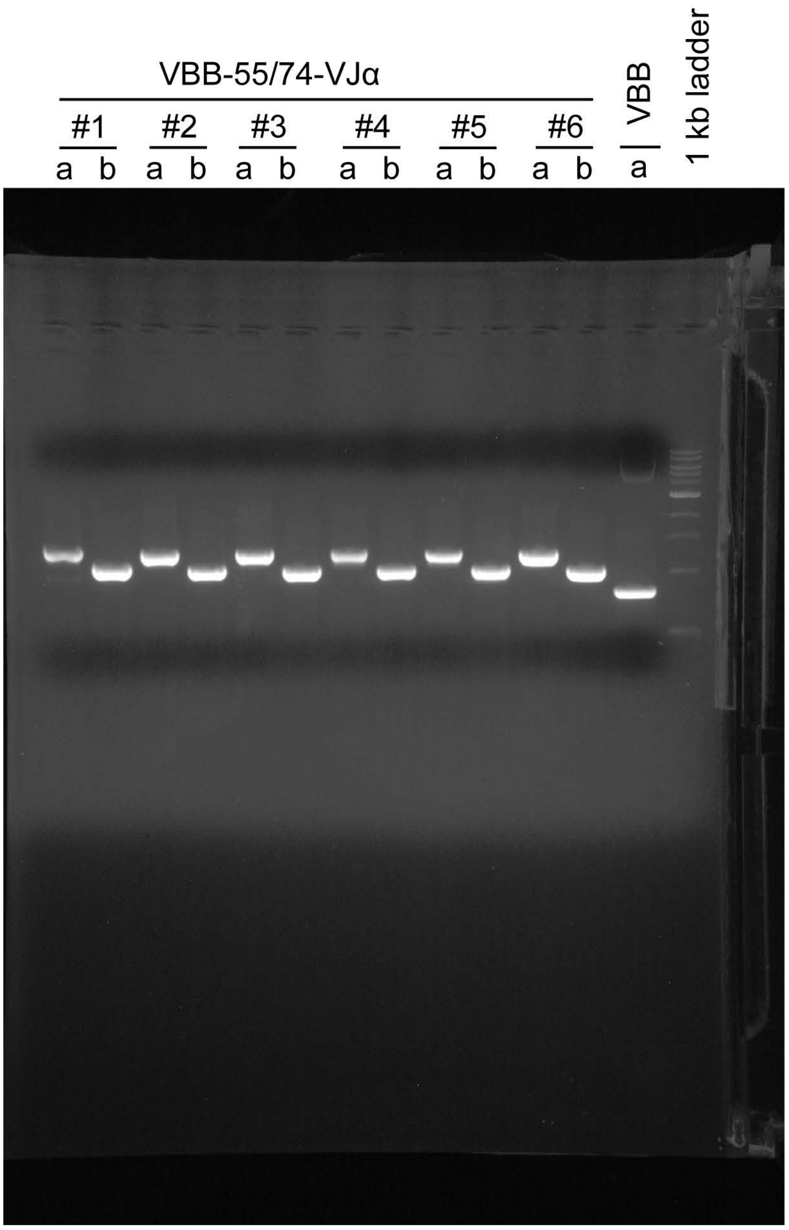

Raw gel image Fig 3A (right pannel)

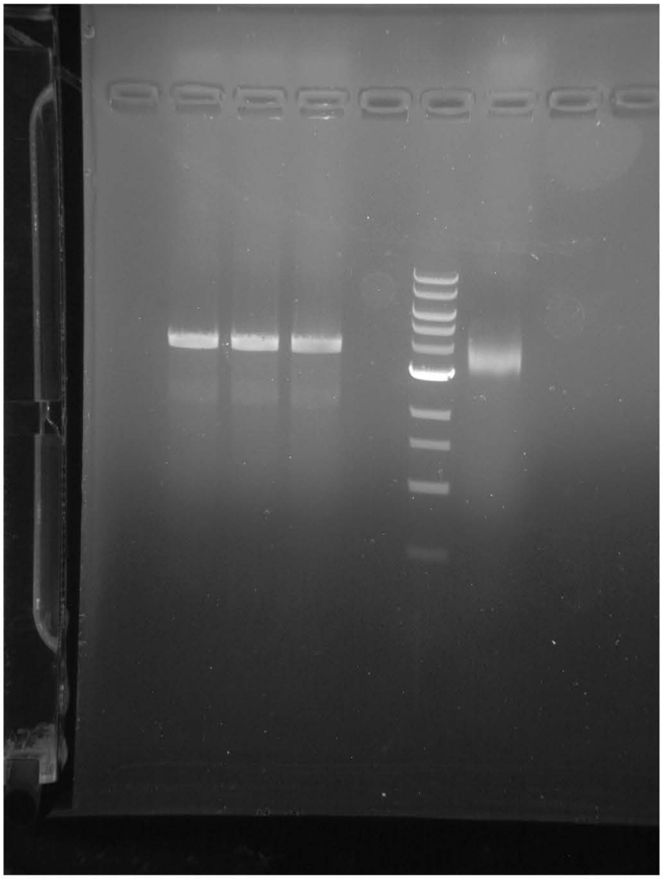

Raw gel image Fig 3B (left pannel)

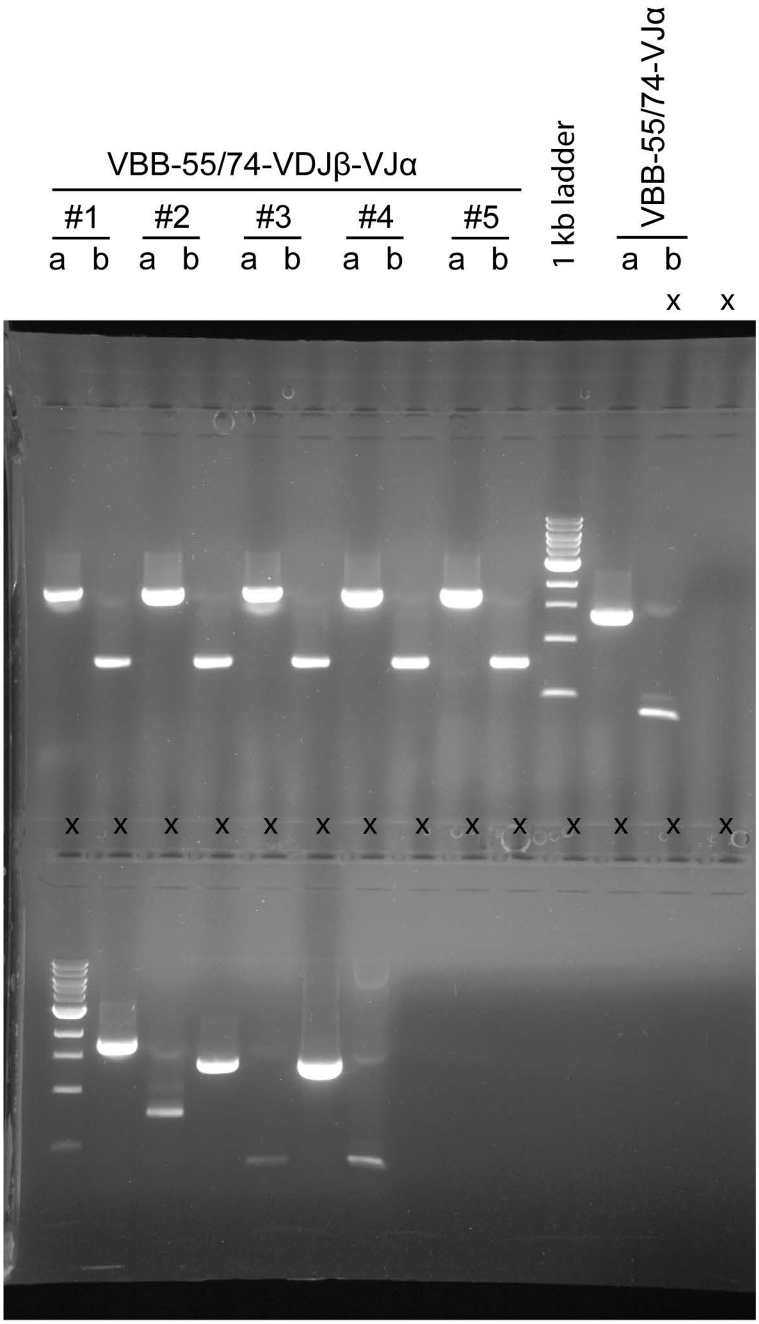

Raw gel image Fig 3B (right pannel)

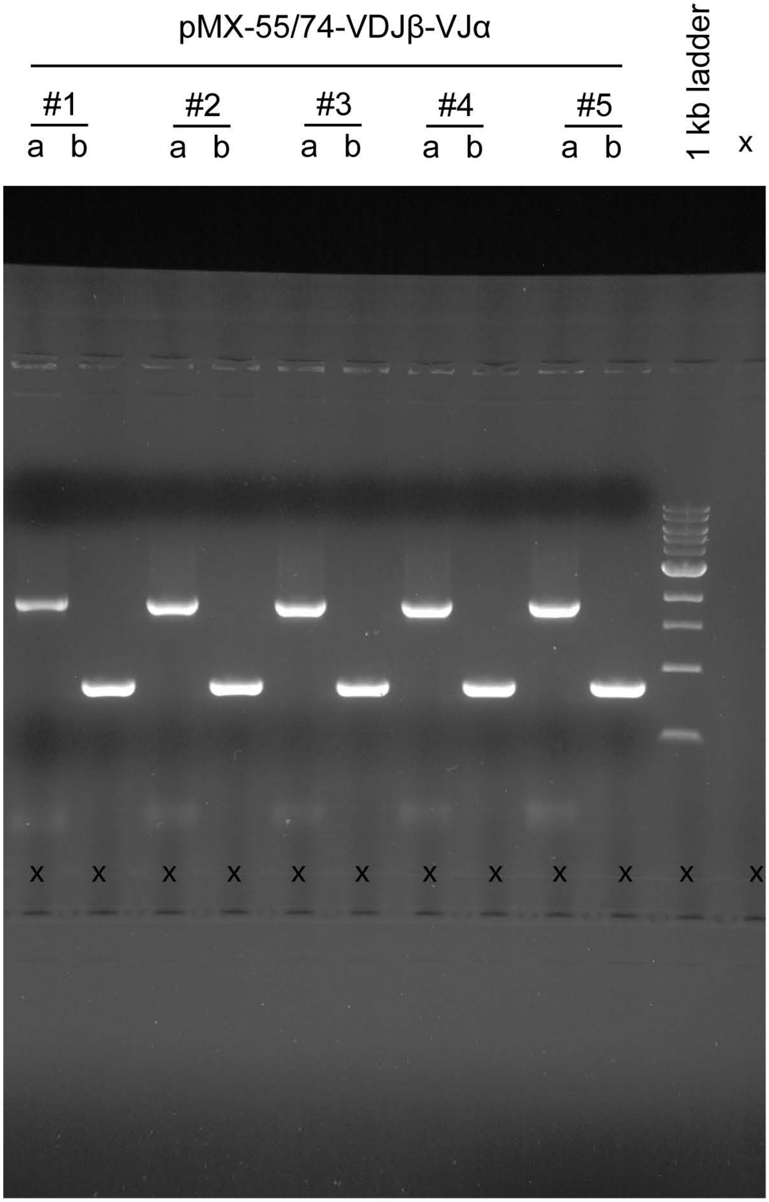

Raw gel image Fig 3C

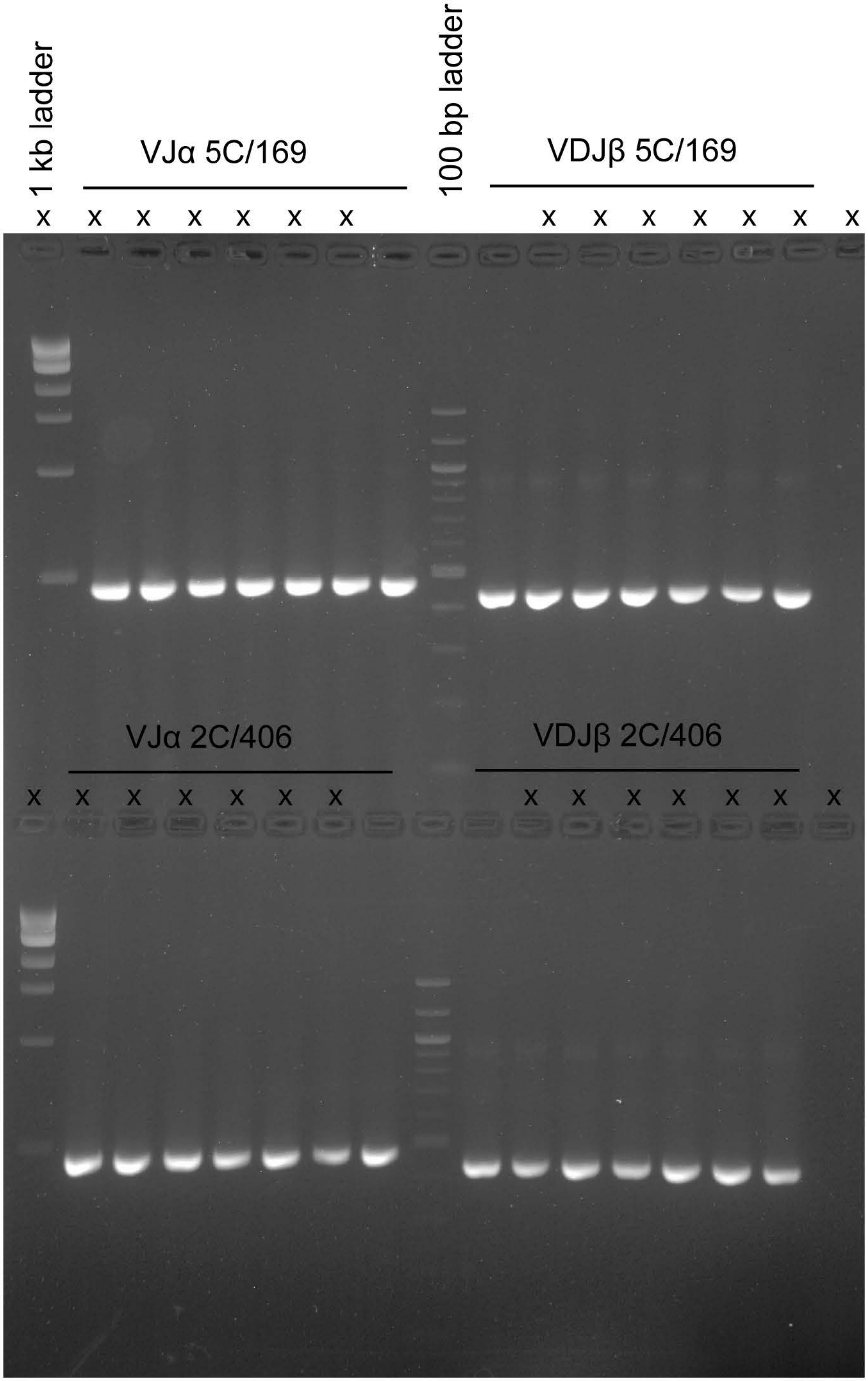

Raw gel image S1 FigB

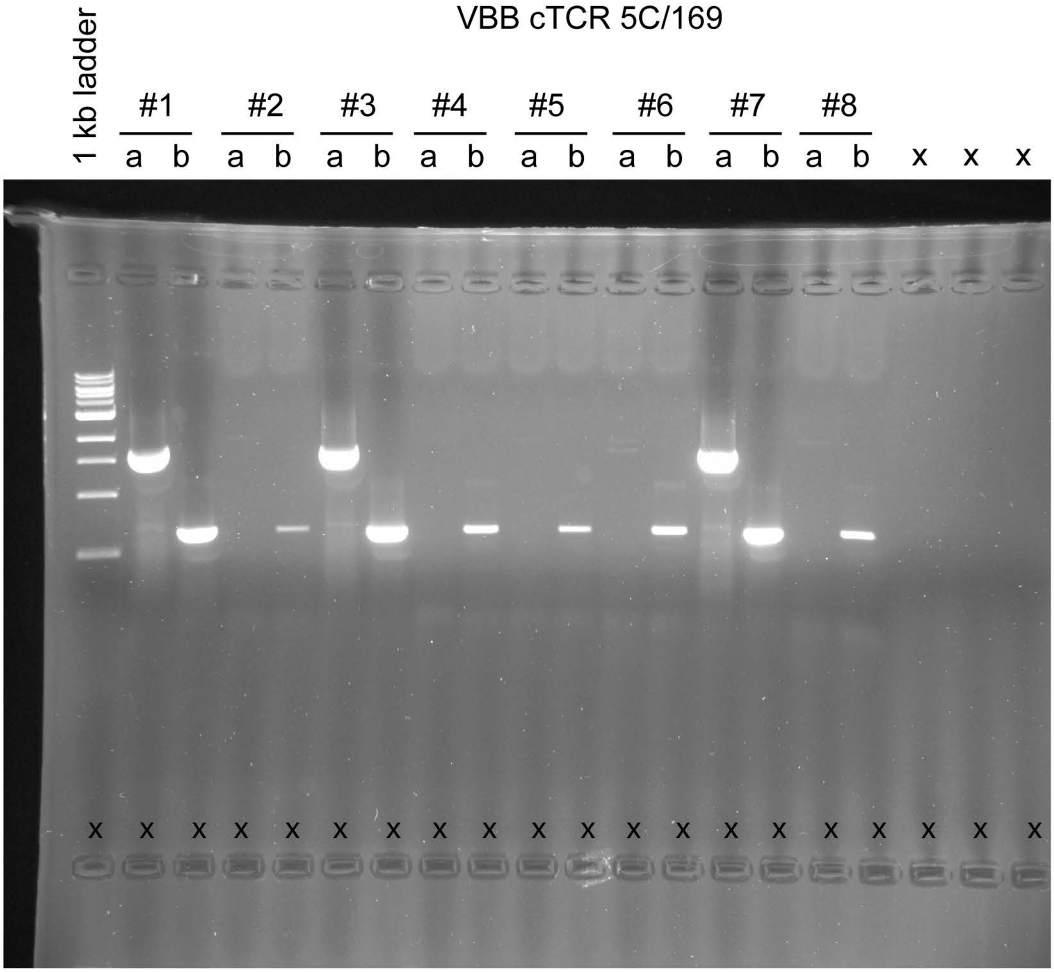

Raw gel image S1 FigC (upper panel)

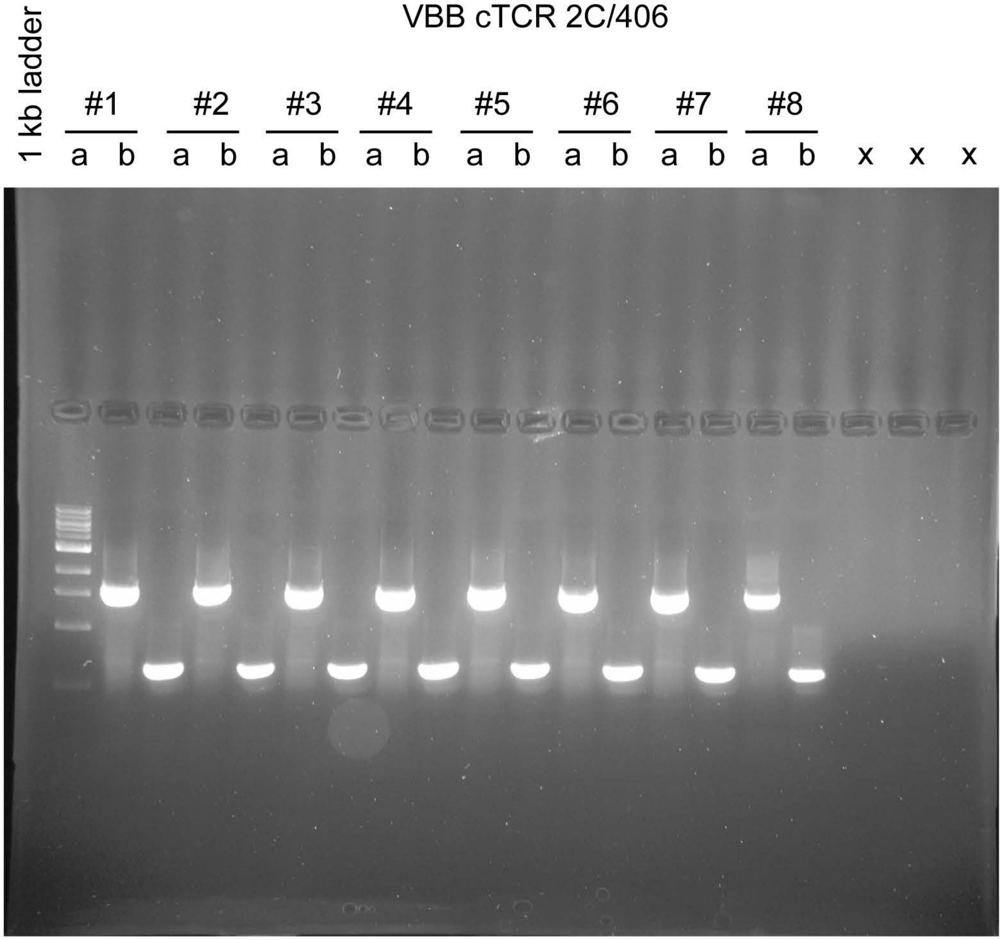

Raw gel image S1 FigC (lower panel)

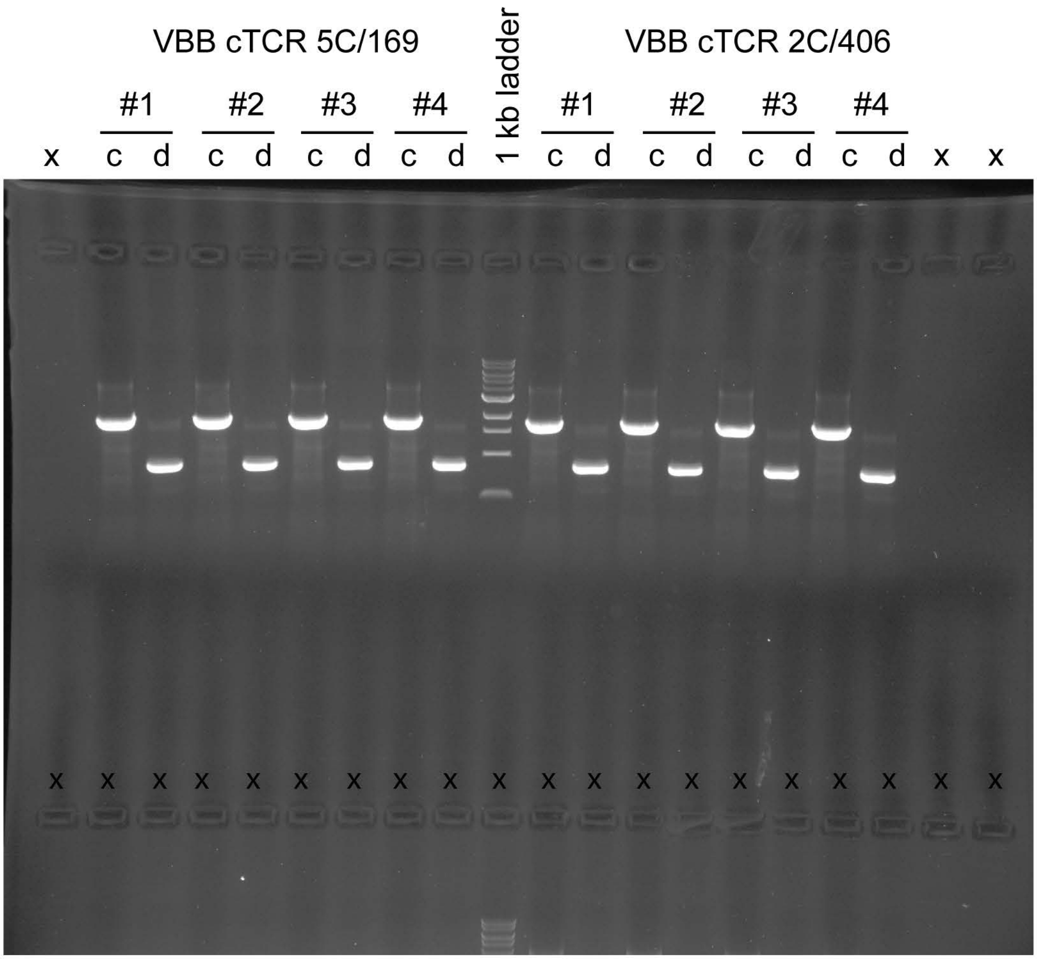

Raw gel image S1 FigD

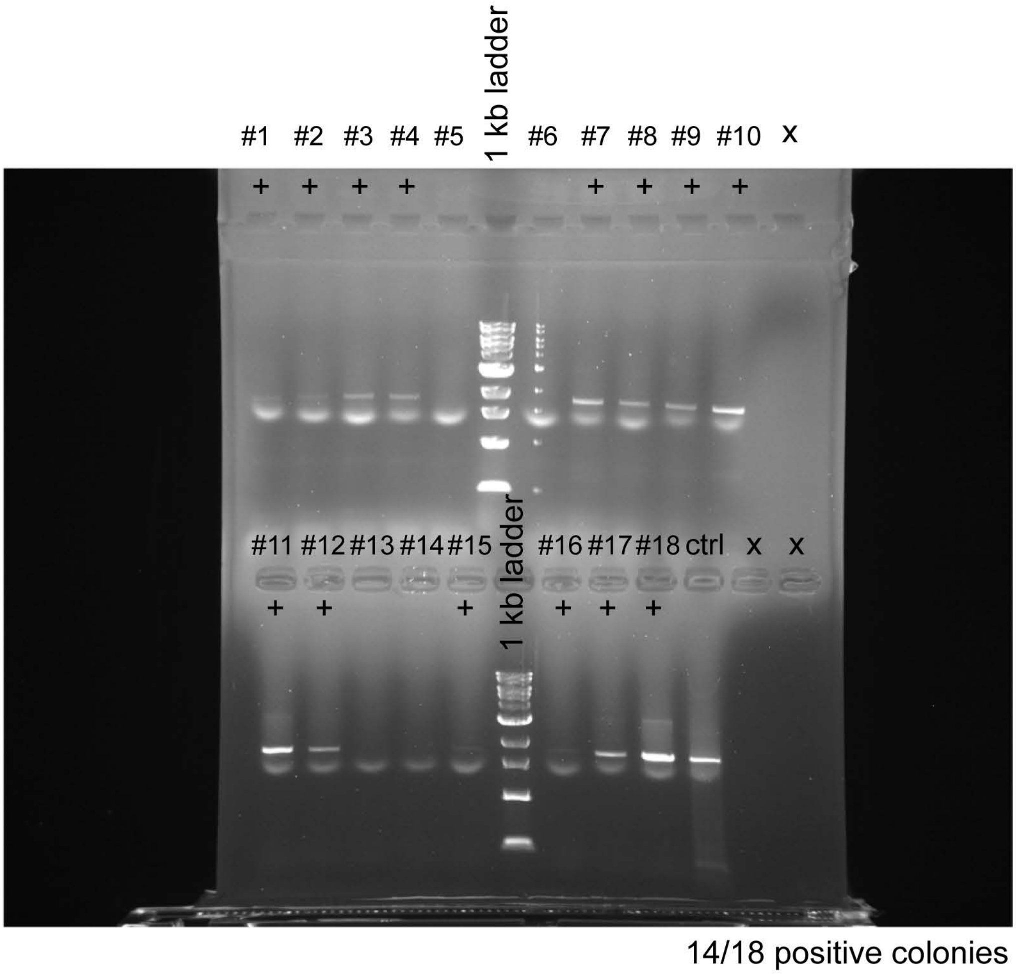

Raw gel image S3 Table: T-cell clone 14/35, colony PCR screen, one-step method, + indicates positive colonies, ctrl indicates VBB-14/35-VDJβ-VJα assembled by the two-step method, primer pair: M13.for, mTRAC.p670.rev

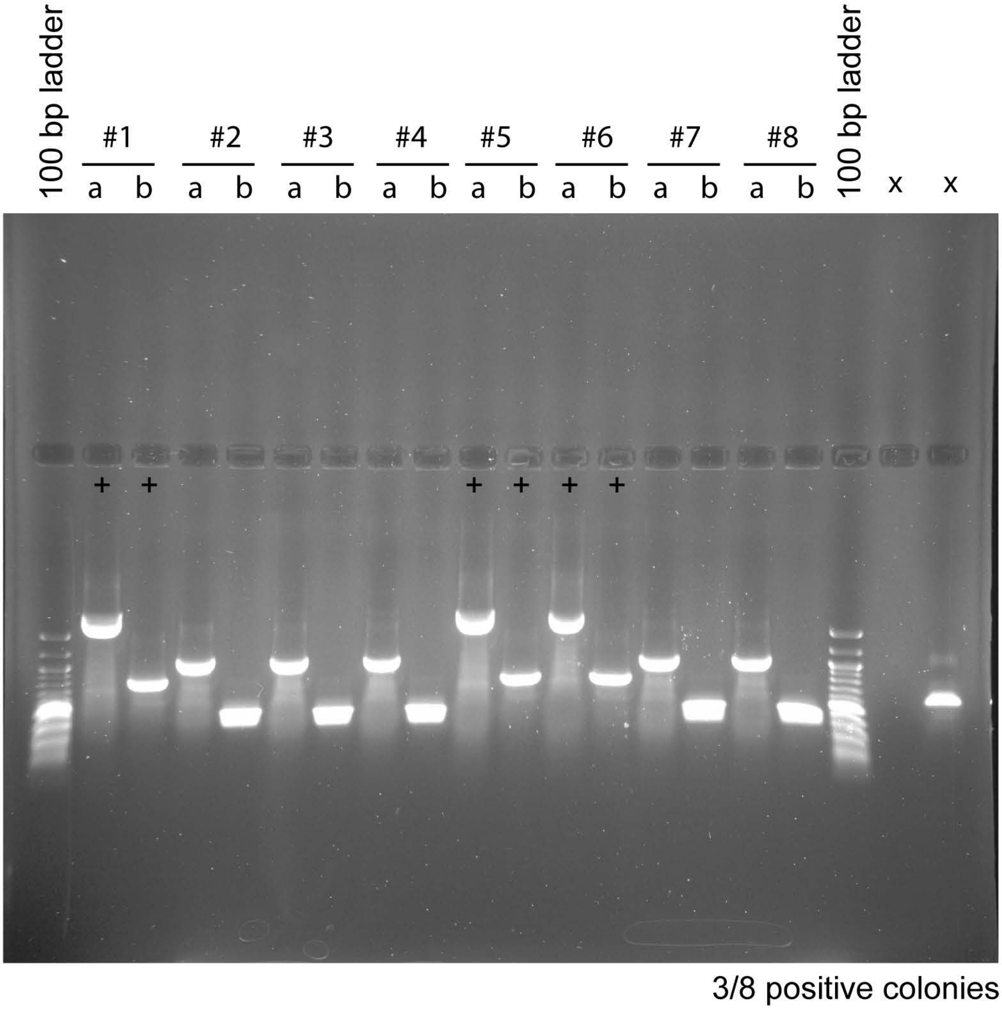

Raw gel image S3 Table: T-cell clone 11C/25, colony PCR screen, one-step method, + indicates positive colonies, primer pair a: M13.for, mTRAC.p670.rev, primer pair b: M13.for, mTRBC.p618.rev

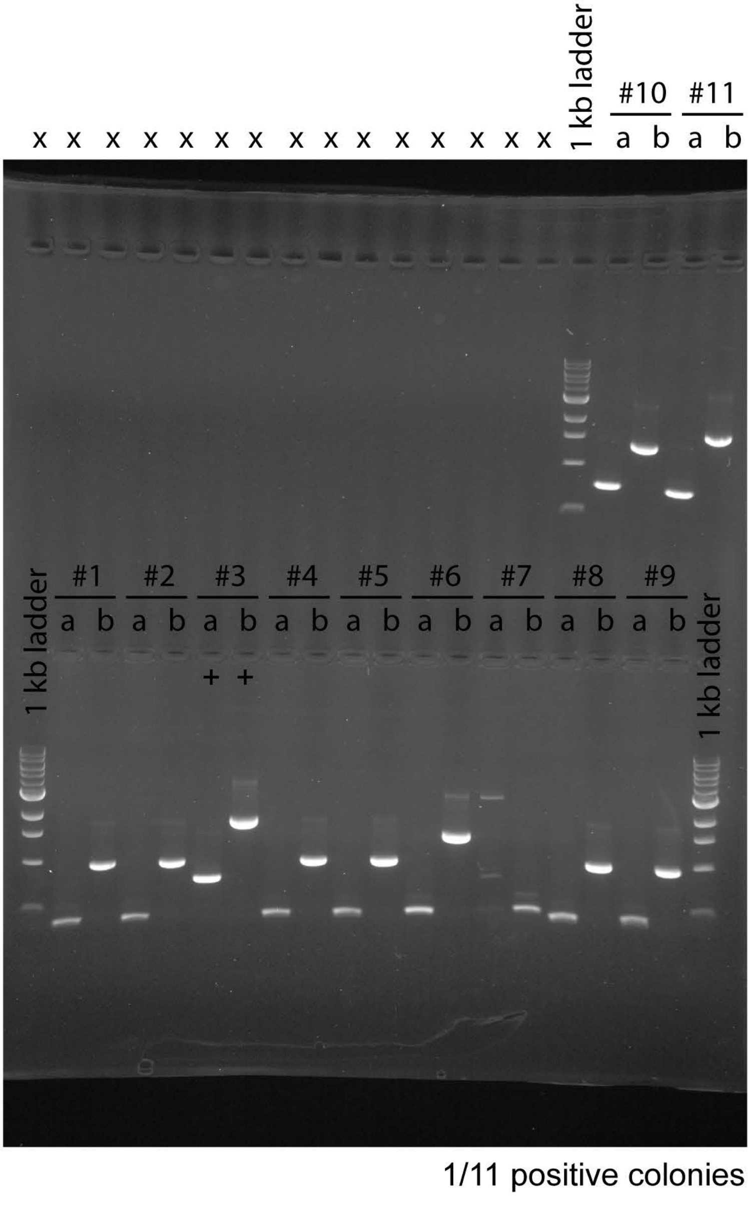

Raw gel image S3 Table: T-cell clone 4/134, colony PCR screen, one-step method, + indicates positive colonies, primer pair a: M13.for, mTRBC.p618.rev, primer pair b: M13.for, mTRAC.p670.rev

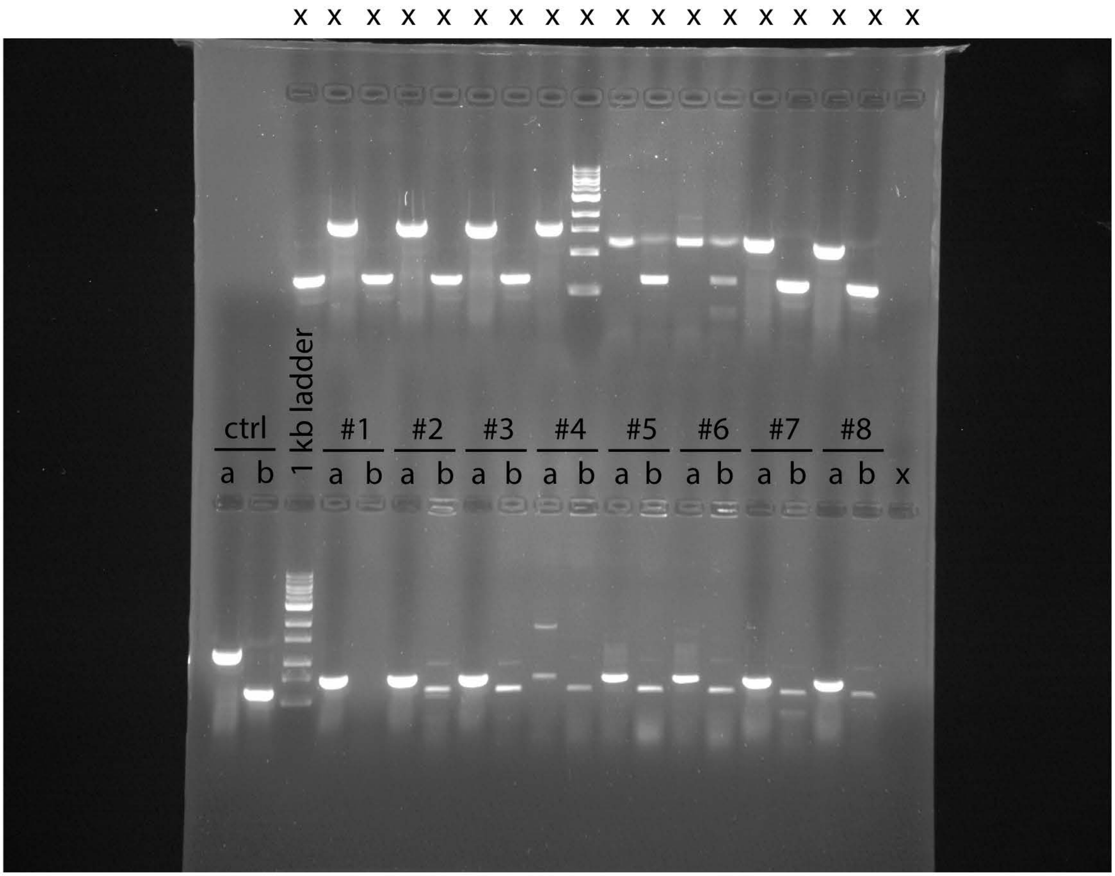

0/8 positive colonies

Raw gel image S3 Table: T-cell clone 4/76 #1-8, colony PCR screen, one-step method, + indicates positive colonies, ctrl indicates #3 of T-cell clone 4/134 assembled by the one-step method, primer pair a: BsaI_TRBV9*02.for, mTRBC.p618.rev, primer pair b: BsaI_TRBV9*02.for, mTRAC.p670.rev

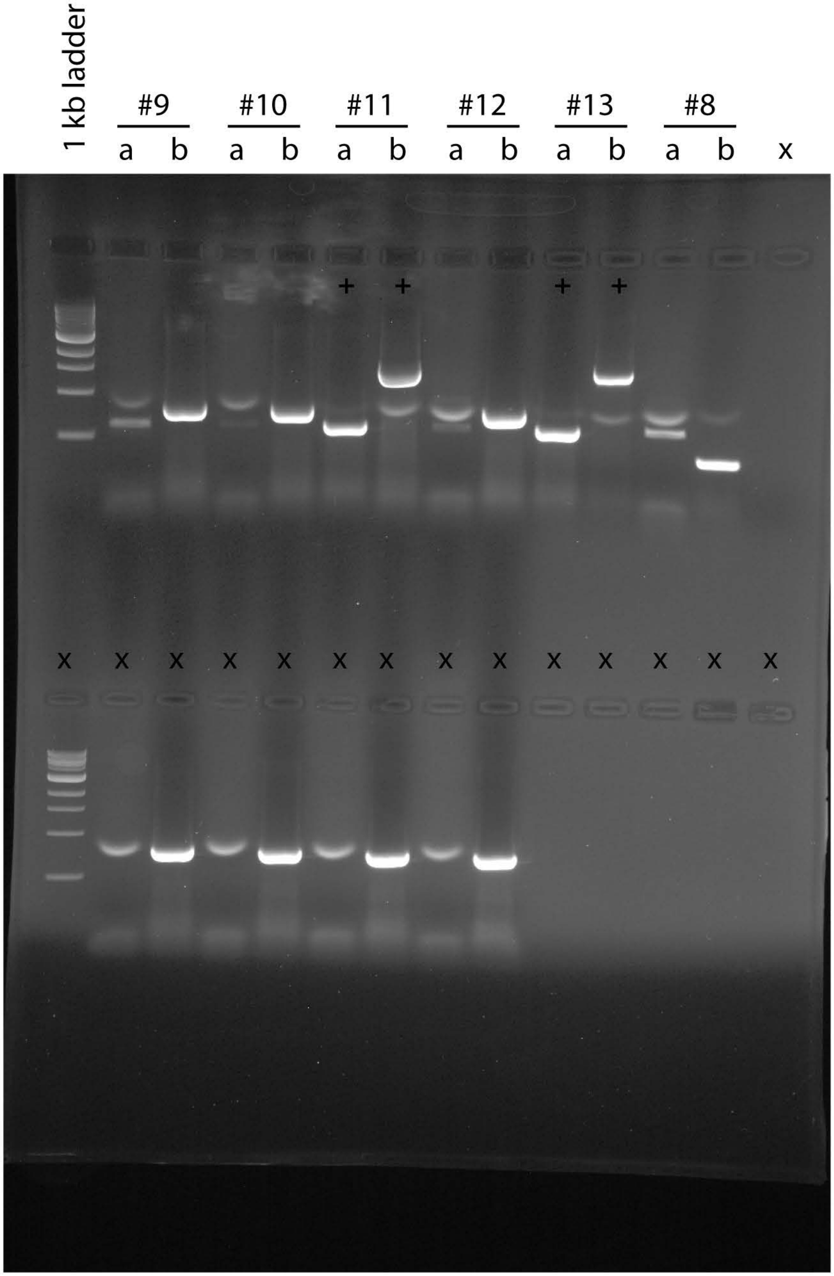

2/13 positive colonies

Raw gel image S3 Table: T-cell clone 4/76 #9-13, colony PCR screen, one-step method, + indicates positive colonies,primer pair a: M13.for, mTRBC.p618.rev, primer pair b: M13.for, mTRAC.p670.rev

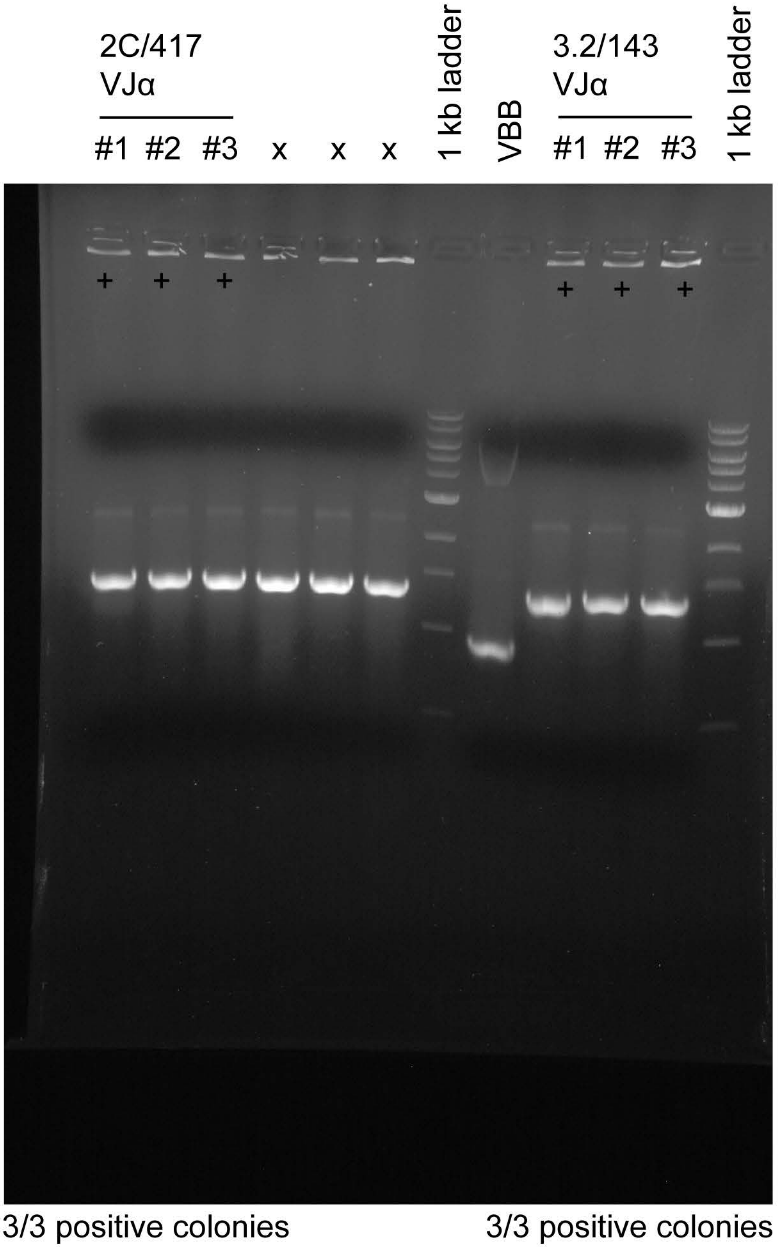

Raw gel image S3 Table: T-cell clone 2C/417 and 3.2/143, colony PCR screen, two-step assembly, + indicates positive colonies, VBB-VJα, primer pair: M13.for, mTRAC.p670.rev

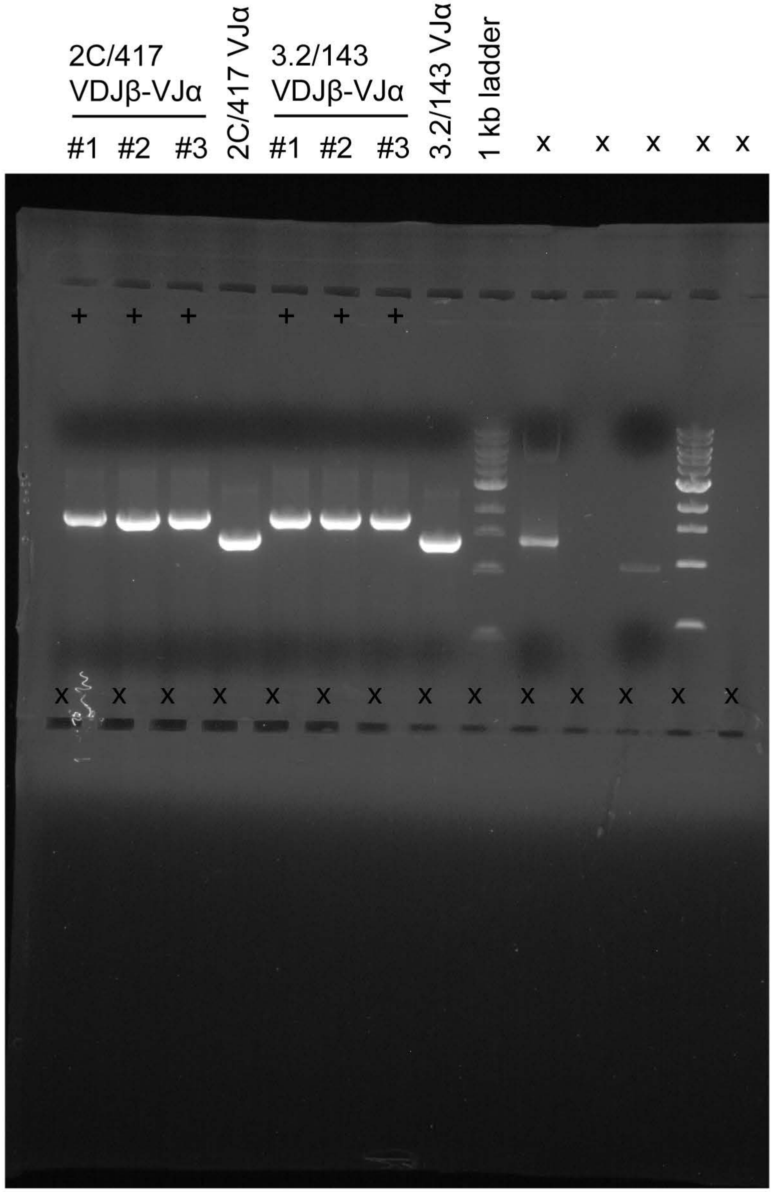

3/3 positive colonies 3/3 positive colonies

Raw gel image S3 Table: T-cell clone 2C/417 and 3.2/143, colony PCR screen, two-step assembly, + indicates positive colonies, VBB-VDJβ-VJα, primer pair: M13.for, mTRAC.p670.rev

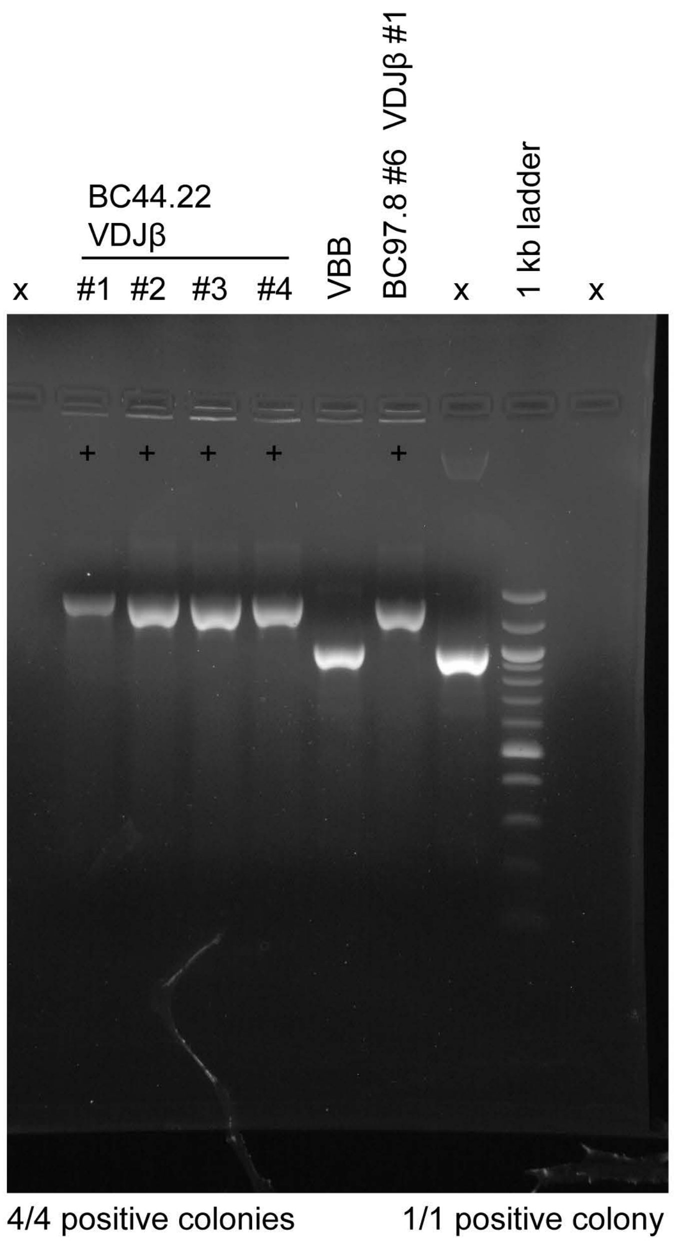

Raw gel image S3 Table: T-cell clone BC44.22 and BC97.8#6, colony PCR screen, two-step assembly, + indicates positive colonies, VBB-VDJβ, primer pair: M13.for, mTRAC.p670.rev

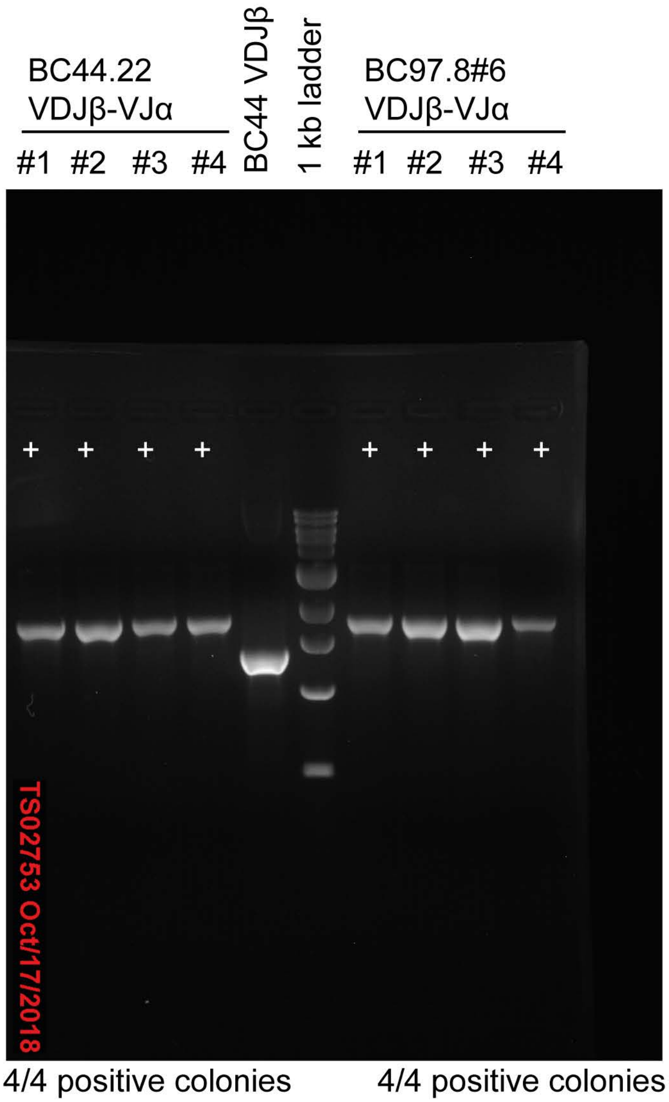

Raw gel image S3 Table: T-cell clone BC44.22 and BC97.8#6, colony PCR screen, two-step assembly, + indicates positive colonies, VBB-VDJβ-VJα, primer pair: M13.for, mTRAC.p670.rev

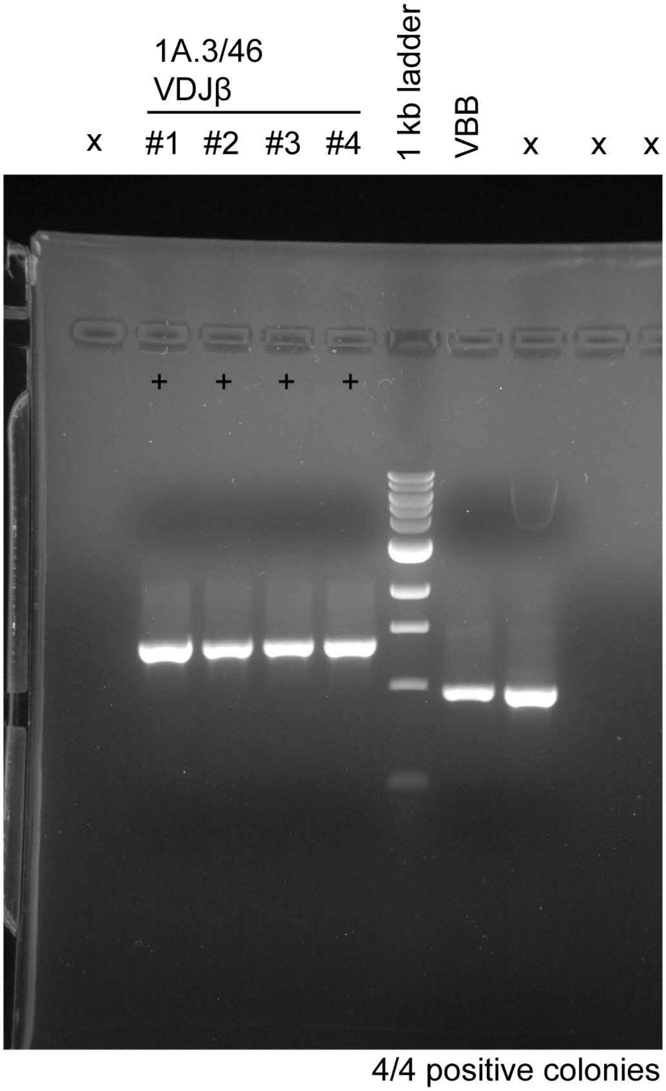

Raw gel image S3 Table: T-cell clone 1A.3/46, colony PCR screen, two-step assembly, + indicates positive colonies, VBB-VDJβ, primer pair: M13.for, mTRAC.p670.rev

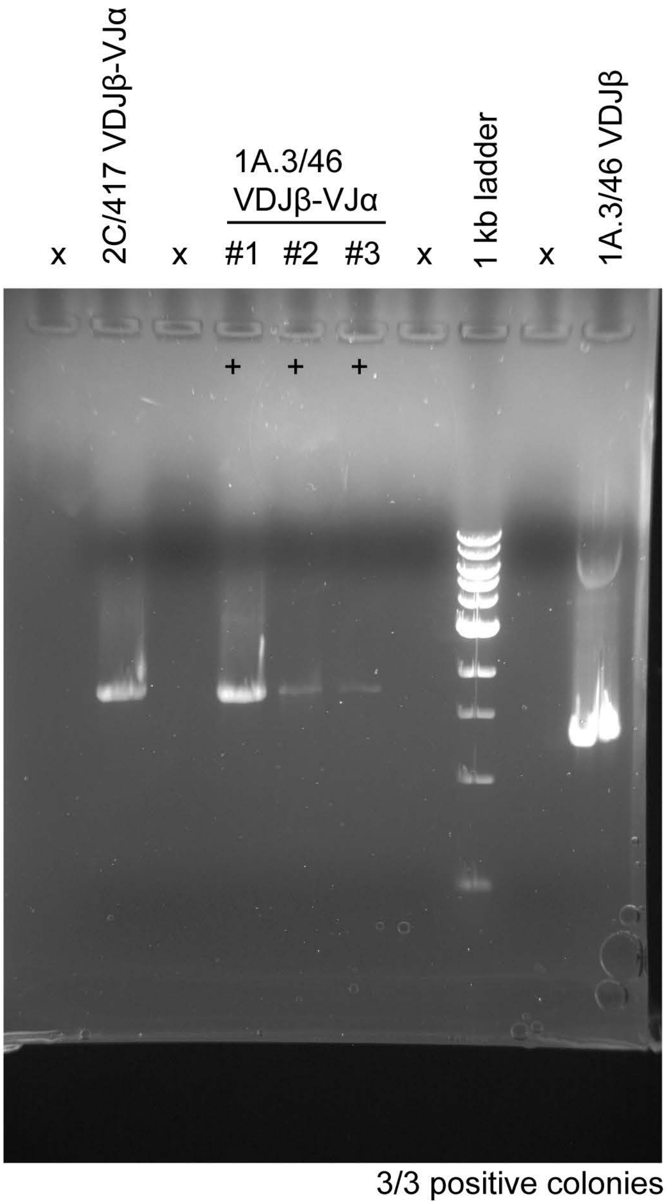

Raw gel image S3 Table: T-cell clone 1A.3/46, colony PCR screen, two-step assembly, + indicates positive colonies, VBB-VDJβ-VJα, primer pair: M13.for, mTRAC.p670.rev

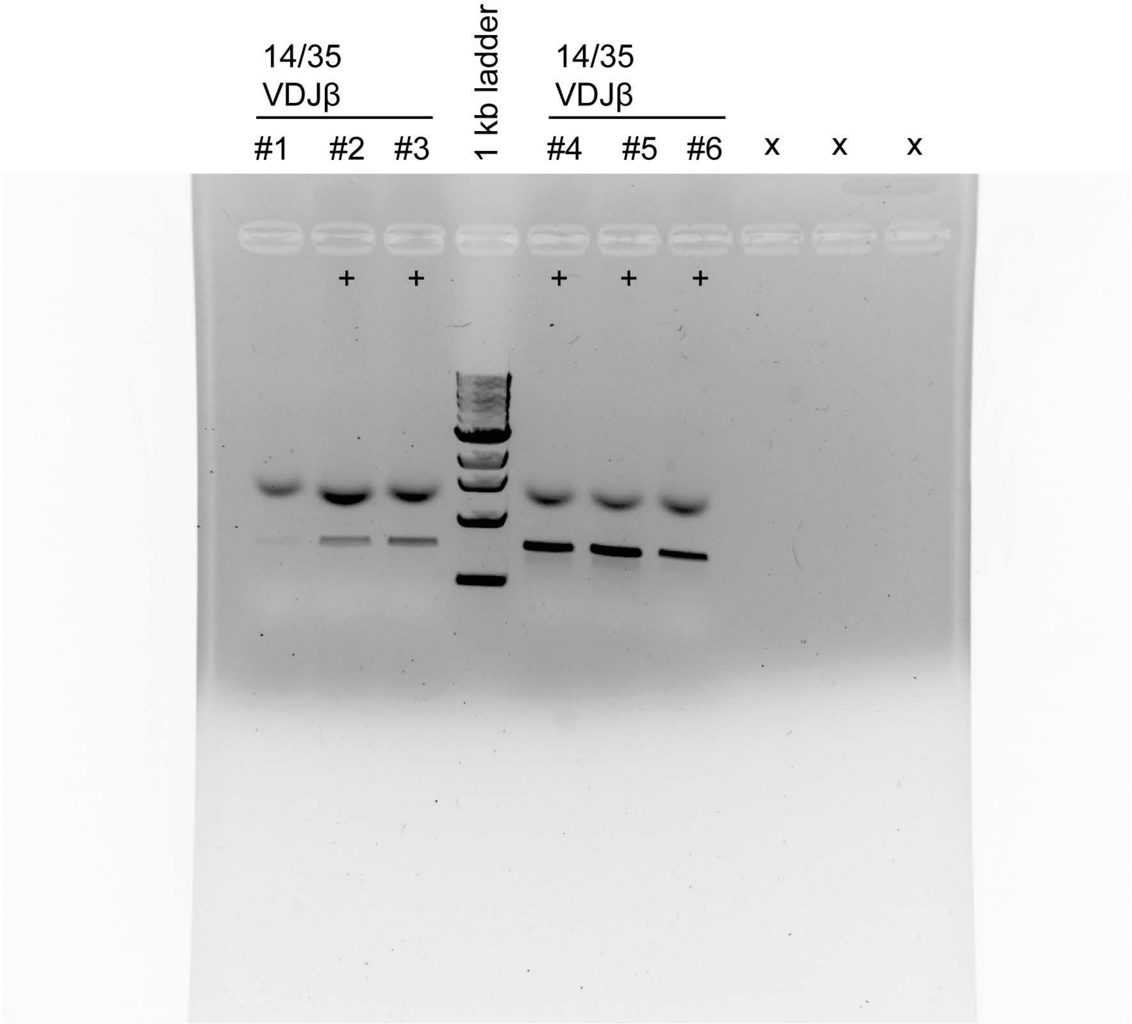

5/6 positive colonies

Raw gel image S3 Table: T-cell clone 14/35, colony PCR screen, two-step assembly, + indicates positive colonies, VBB-VDJβ, primer pair: M13.for, mTRBC.p618.rev

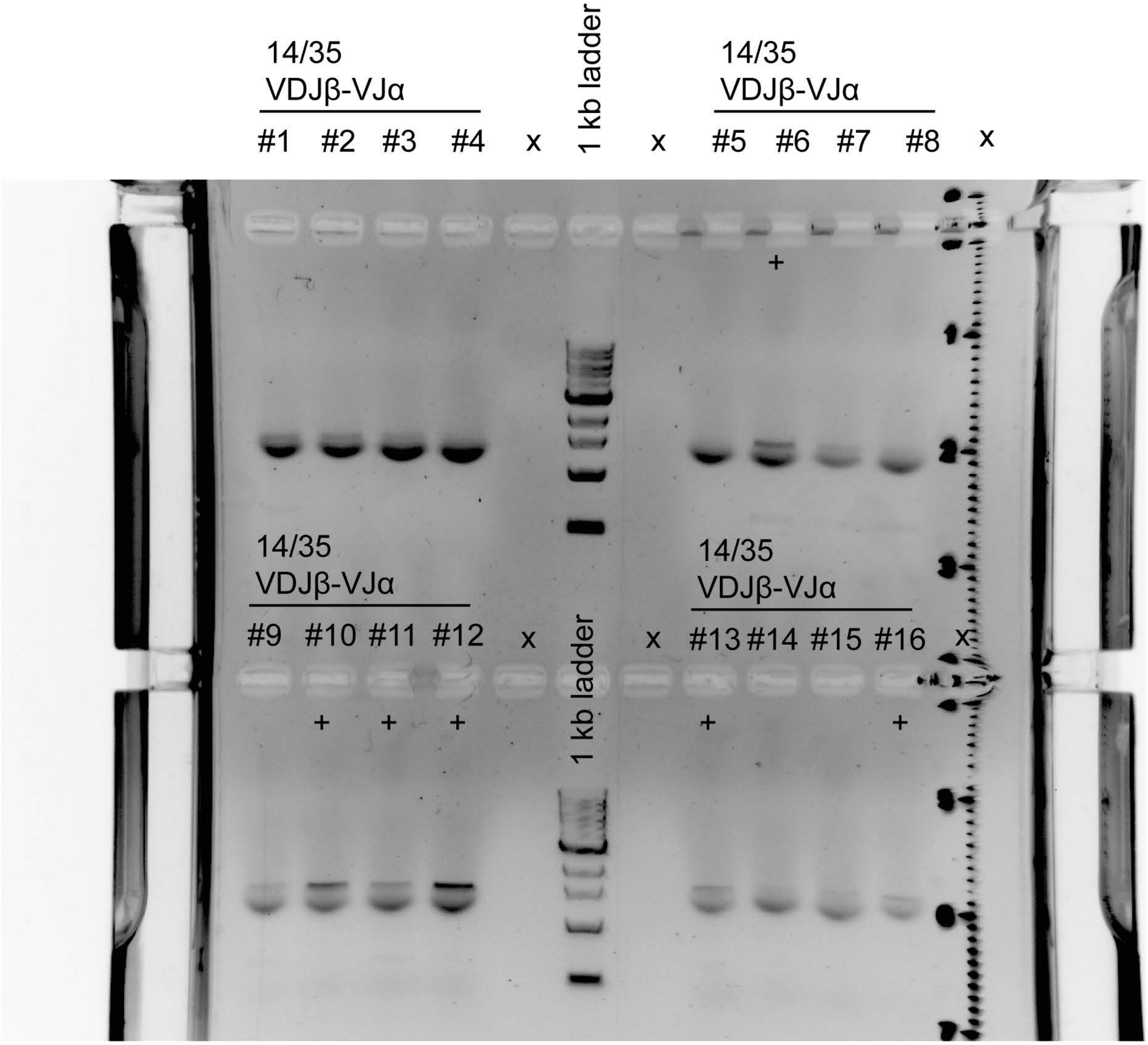

6/16 positive colonies

Raw gel image S3 Table: T-cell clone 14/35, colony PCR screen, two-step assembly, + indicates positive colonies, VBB-VDJβ-VJα, primer pair: mTRBC.p479.for (5‘-GCCGAGATCGCCAACAAGCAG-3‘), M13.rev (5‘-CAGGAAACAGCTATGACC-3‘)

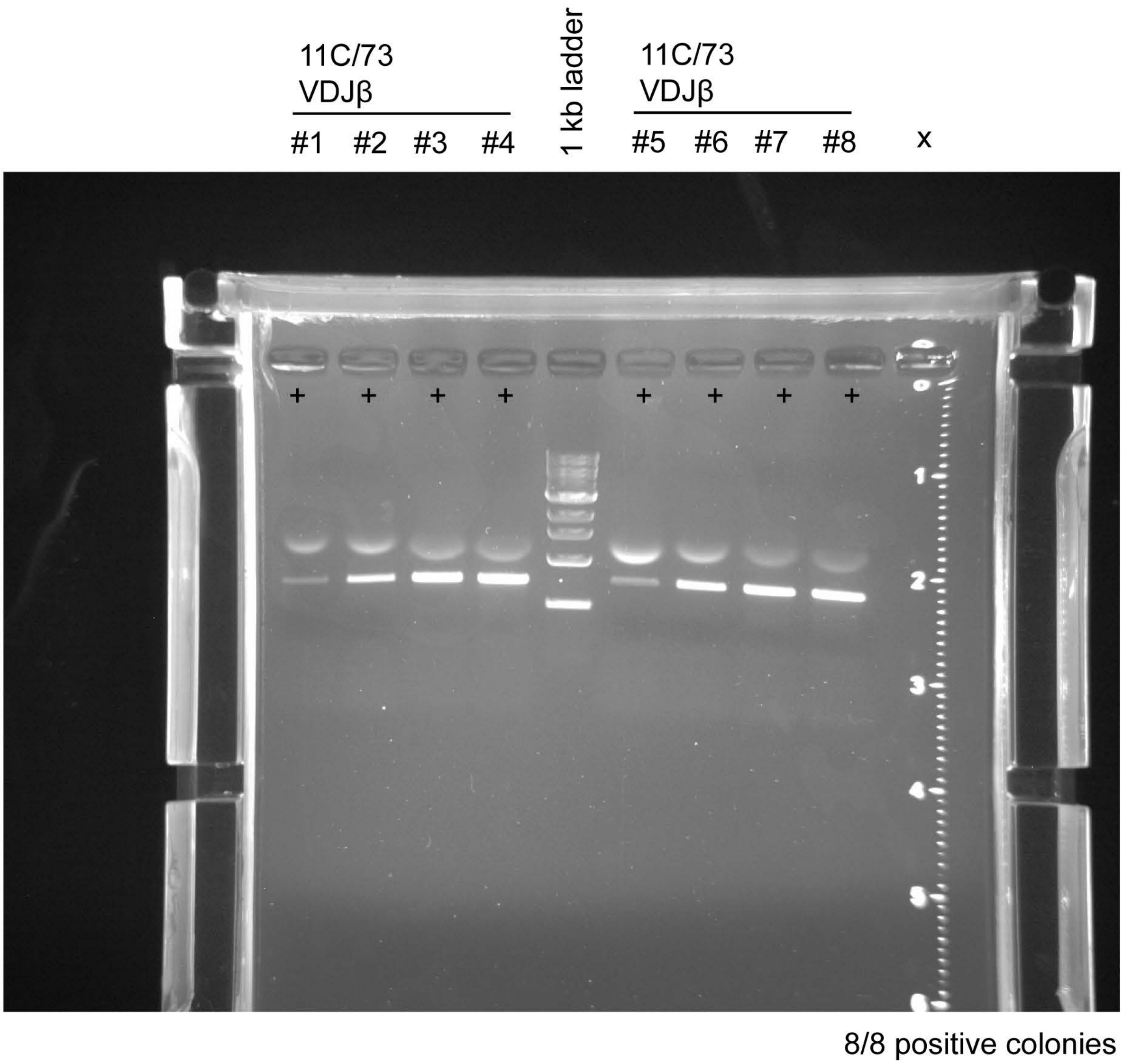

Raw gel image S3 Table: T-cell clone 11C/73, colony PCR screen, two-step assembly, + indicates positive colonies, VBB-VDJβ, primer pair: M13.for, mTRBC.p618.rev

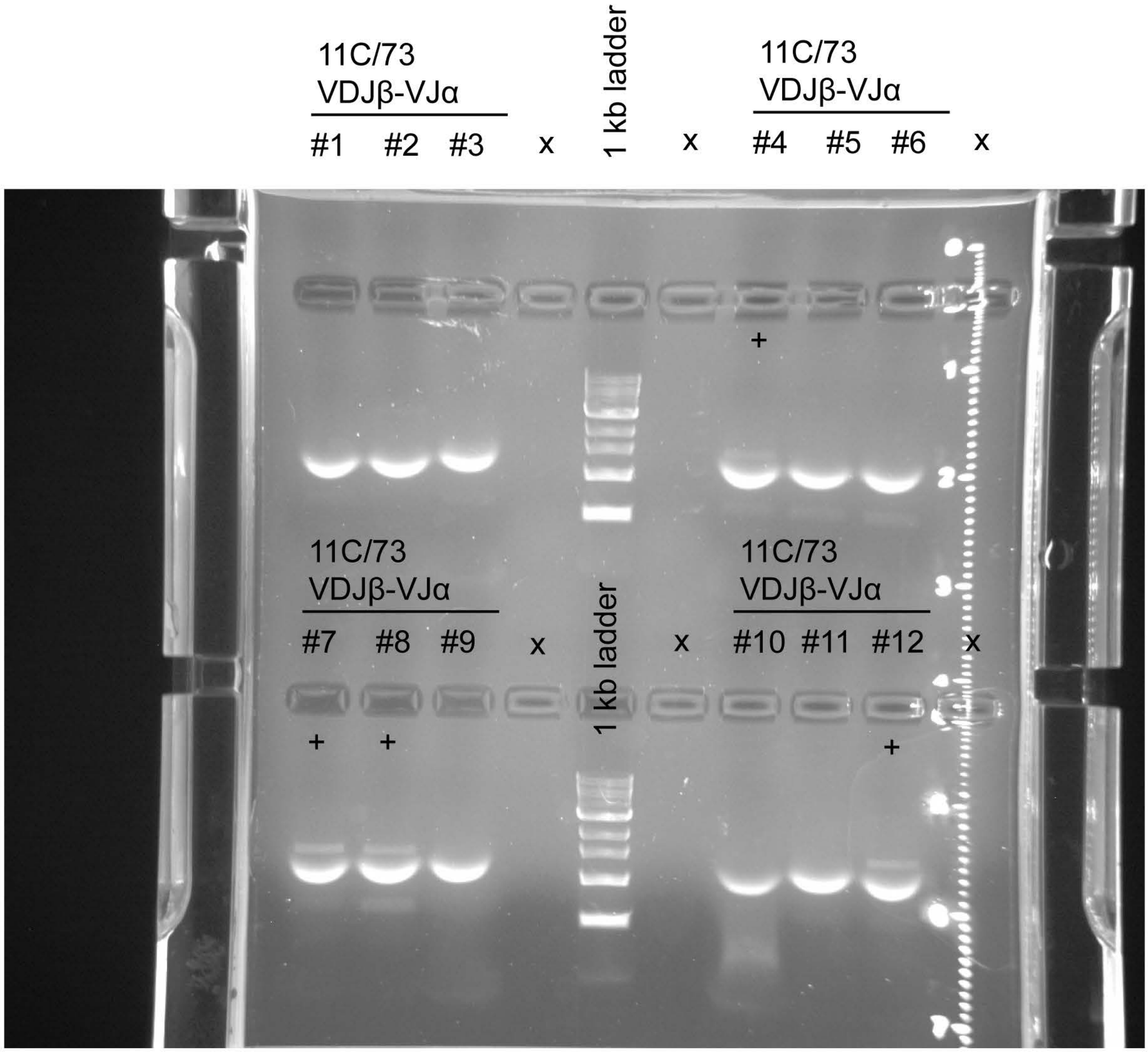

4/12 positive colonies

Raw gel image S3 Table: T-cell clone 11C/73, colony PCR screen, two-step assembly, + indicates positive colonies, VBB-VDJβ-VJα, primer pair: mTRBC.p479.for, M13.rev
